# Household dysfunction and child outcomes in the Nordic countries: A bibliometric analysis

**DOI:** 10.1177/14034948251336851

**Published:** 2025-05-24

**Authors:** Rebecca L. Radlick, Alice Milivinti, Håvard T. Rydland, Ingrid R. Lundeberg, Kristin G. Askeland

**Affiliations:** 1 Division of Health & Social Sciences, NORCE Norwegian Research Centre, Bergen, Norway; 2 Department of Correctional Studies, University College of Norwegian Correctional Service (KRUS), Oslo, Norway; 3 Regional Centre for Child and Youth Mental Health and Child Welfare, NORCE Norwegian Research Centre, Bergen, Norway

**Keywords:** Adverse childhood experiences, household dysfunction, mental health, somatic health, labour market, school completion, incarceration, parents, resilience

## Abstract

**Aims:**

This article provides a bibliometric analysis of the literature on adverse childhood experiences (ACEs) related to household dysfunction (parents’ substance abuse, physical or mental illness, death, criminality, and divorce/separation) in five Nordic countries. We identify: 1) main patterns and characteristics of the literature on household dysfunction ACEs and child outcomes; 2) highlight research gaps, topics and approaches for future inquiry on these ACEs.

**Methods:**

A systematic search for peer-reviewed articles published from 1998 to 2022 in English was conducted in seven databases. Information from the articles was extracted using a coding matrix that included variables related to country, specific household dysfunction ACE(s) occurring before 18 years, child outcome(s), method, data source(s) and whether resilience or protective factors were assessed. Bibliometric analyses were used to summarize the literature patterns.

**Results:**

A total of *N*=5003 publications were identified and *n*=342 publications were included in the analysis. *n*=112 publications studied two or more ACEs of interest. Divorce/separation was the most common individual ACE (*n*=97), whereas parental criminality was the least common (*n*=9). *n*=197 publications studied child mental health outcomes, whereas educational (*n*=41) and labour market (*n*=11) outcomes were less represented. Few (*n*=36) studies included protective factors.

**Conclusions:**

Our findings suggest a notable increase in research on household dysfunction adversities in the Nordic countries over the past two decades, focusing mainly on health-related outcomes. Future research should investigate less represented adversities, functional outcomes and protective factors. Interdisciplinary and new methodological approaches can provide fresh insights into this public health challenge.

## Introduction and background

Adverse childhood experiences (ACEs) are potentially traumatic or stressful events occurring during childhood or adolescence [1] that can have short- and long-term consequences for health, health behaviours, mortality [2,3], academic skills [4], executive function [5] and quality of life [6].

The ACEs addressed in the literature are heterogenous [7]. Traditionally, specific ACEs have been categorized into one of two groups: related to *child maltreatment*, which encompasses various forms of child abuse and neglect, and *household dysfunction*, which includes adversities in the family environment and is linked to the parents [1]. More recently, community-level adversities such as school bullying, natural disasters or wartime experiences have also been studied as ACEs [8,9]. The *household dysfunction* ACEs – including parental separation or divorce, substance abuse, death, physical or mental illness, and incarceration – are particularly relevant. They are not only among the most common [1,10], but also critically shape children’s development, health and well-being owing to the pivotal role of parents in their lives [11]. Although ACEs are a global public health concern, their incidence and impacts can vary by context, which motivates our bibliometric analysis of Nordic research on household dysfunction ACEs, where the comprehensive welfare systems could mitigate the negative consequences of household dysfunction adversities.

Household dysfunction ACEs can disrupt the quality and consistency of parental support, profoundly affecting children’s immediate environment and long-term development, as parents typically serve as the primary source of support and behavioural modelling for their children [12,13]. Research has demonstrated that maternal mental illness is associated with negative parenting behaviour and child disengagement [14]. Subsequent studies have further investigated the complex challenges faced by children in this context, revealing multifaced family dynamics including emotionally and/or physically unavailable parents, financial strain, need for additional adult support [15], challenges in parent–teacher relationships [16], and role reversal, where children assume parental responsibilities [17].

ACEs are common and often co-occur [1,18]. One systematic review and meta-analysis of 10 European studies found that 23% of individuals experienced one ACE, and 18.7% two or more ACEs [19]. Many ACE studies neither justify their inclusion/exclusion criteria for specific adversities [20] nor differentiate their relative impact, often weighing all adversities equally. While this cumulative approach highlights overall exposure effects and acknowledges co-occurrence of adverse events [20], it limits the understanding of the effects of specific adversities and the mechanisms which lead to outcomes. International reviews on ACE outcomes predominantly focus on general physical and mental health [3,21,22] or specific diseases or disorders [23–26]. Although the association between ACEs and mental health is well established, even after adjusting for familial confounding [27], fewer studies have investigated functional outcomes, such as labour market participation [28], or socio-relational outcomes. While this pattern is evident in the broader ACE literature, there is currently no overview of research on household dysfunction ACEs and their outcomes. A previous bibliometric analysis [29] provided insights into the international ACEs research landscape, but did not explore regional patterns, the specific links between ACE exposure and outcomes or focus exclusively on household dysfunction.

Nordic countries are often recognized as model systems in social welfare and public health [30], with policies and substantial family support investments that could mitigate household dysfunction adversities. Ranking among the top nine OECD countries for family-specific financial support as percentage of GDP [31], these nations offer a unique context for studying childhood adversity. As most ACE research has been conducted in the United States [3,7,22] with no literature studies focusing exclusively on household dysfunction in the Nordic region, our analysis aims to address this research gap.

Given the prevalence of and well-established relationship between childhood adversities and negative short- and long-term outcomes, identifying protective factors or interventions that contribute to positive development is crucial. Not all individuals exposed to ACEs experience adverse outcomes [32], suggesting the development of *resilience* – a process where individuals withstand and recover from adversities, showing positive adaptation despite their risk exposure [33]. Protective factors contributing to resilience include *individual-level* characteristics, *family* support and *external environmental* support [33–35]. A close relationship with a parent or other supportive adult has been highlighted as the most important protective factor in childhood and adolescence [34,36]. When household dysfunction disrupts parental support, identifying alternative protective factors becomes essential [37]. Understanding the relationship between household dysfunction and child outcomes, along with protective factors that facilitate resilience, is crucial for developing evidence-based interventions for affected children and families.

The current study aims to examine household dysfunction ACEs research in the five Nordic countries from 1998 to 2022 using bibliometric methods. Household dysfunction ACEs include parental: substance abuse, incarceration/criminality, divorce, separation, or parents no longer living together (hereafter referred to as ‘divorce/separation’), mental or physical illness, and death. We performed systematic database searches, screenings and data extraction. We describe the publication trends in the literature by individually analysing household dysfunction ACEs and child outcomes, as well as their intersections, interplay between adversities and outcomes, and type of analysis (methods, data source, research design) over time. In addition to the overview of the literature, we include a section on the role of resilience and protective factors.

## Aim

Our aim is to provide a bibliometric analysis of the Nordic research literature related to household dysfunction ACEs. We identify: 1) the main patterns and characteristics of the literature on household dysfunction ACEs and child outcomes in the Nordic countries, also longitudinally; 2) the research gaps, topics and approaches for future inquiry on these ACEs.

## Method

We combine traditional processes for manually and systematically searching, collecting and selecting publications (specifying aims, systematically searching for scientific literature, screening and selection based on pre-determined inclusion and exclusion criteria) with bibliometric analyses to summarize and present our results [38,39]. Bibliometric analyses allow for the exploration and quantitative analysis of large volumes of scientific data, elucidating main themes and patterns and highlighting research gaps in a specific field, often with numerous visualizations [39]. This differs from traditional systematic or scoping review approaches, which present and synthesize results from individual studies and report on biases and certainty of evidence [40].

Systematic searches were performed in April 2022 in seven electronic databases (Web of Science, PsycInfo, Medline, Embase, ERIC, CINAHL and Scopus) using the approaches outlined by Page et al. [40]. The authors of a previous international bibliometric analysis searched only for the phrases ‘adverse childhood experience’ or ‘adverse childhood event’ [29], limiting their review and excluding literature on adversities from disciplines which do not engage with this term. Thus, two broad searches were conducted, with retrieved citations combined. The first explicitly focused on ACE literature. This entailed a Boolean search string encompassing the five Nordic countries and variations on the term ‘adverse childhood experiences’ (for example: ‘adverse childhood events’, ‘childhood adversities’). The second search focused on specific household dysfunction adversities. This included variations on the following terms related to parental: incarceration, substance/alcohol abuse, illness/health shocks, and divorce/separation. These searches were combined with variations on the word child (adolescent, youth) and terms used for the five Nordic countries.

We included empirical peer-reviewed research articles published in English and with data from one or more of the Nordic countries (Norway, Sweden, Denmark, Iceland, Finland). We excluded articles published prior to 1998, because this was when the first major ACE study was published [1] and welfare state institutions, such as child protective services, have had reforms over the years. No limits were placed on study design. While we searched for the term ‘incarceration’ we also retained all studies that dealt with criminal offences in general. We exclude child abuse/neglect from our ACEs of interest, as our primary focus is on adversities in the household dysfunction category. This has consistently been conceptualized as being separate from child maltreatment [1,41]. Additionally, maltreatment may be particularly detrimental to children, with stronger, independent effects on later outcomes compared with other types of dysfunction, suggesting that it may be useful to study these types of adversities separately [42]. While other adversities are important, we felt that a narrower focus on these specific ACEs would allow for a more in-depth exploration of the parental role in adverse childhood experiences, while ensuring that the study remains within a manageable scope, as the literature on abuse/neglect is extremely comprehensive.

To gain a comprehensive overview of the Nordic household dysfunction ACE literature, we did not include search terms related to resilience at this stage of the process. We expected only a small number of the studies to assess resilience and protective factors, and including these terms in the search would have greatly limited the scope of the database search. The articles identified in the overall search were screened for information on resilience and protective factors at the full-text screening stage.

A total of 5003 publications were retrieved in the database search. After removing duplicates, publications not in English, those which were not peer reviewed articles and those published before 1998, 1314 articles remained. These abstracts were divided and independently screened by three of the co-authors (IL, HR, RLR) using Rayyan [43], a screening tool for systematic reviews. At this stage, we placed no specific limitations on methodology, types of adversities (other than that they should be related to the parents and occur before the age of 18 years) or outcomes (other than that they should be related to the child at any age).

In the full-text screening, we further refined the inclusion criteria as follows: we excluded articles with empirical data from before 1960 and we excluded articles focusing on (epi)genetic explanations. Additionally, given our focus on the Nordic welfare state context, cross-national articles must focus exclusively on two or more Nordic countries and exclude countries outside the Nordic region.

In all, 639 articles were allocated, and the retrieved publications were assessed in full text by all the co-authors. A total of 342 articles met our pre-determined criteria for inclusion in this review. Information from these articles was extracted independently by the assigned co-author using a coding matrix with pre-defined categories that were developed, pilot tested and then further refined in the researcher group. The final version of the coding matrix included variables related to the country, specific household dysfunction ACE(s), outcome(s) for the child, method, data source(s) and whether resilience or protective factors were assessed. For validation, the team independently coded the same seven articles and compared the coding results, discussing any discrepancies to ensure similar coding approaches. During the full text assessment process, any uncertainties in coding were discussed at regular group meetings to ensure coherent coding across the co-authors. Visualizations were created (by AM) using R [44] and the following packages: ggplot2 [45] and ggalluvial [46]. The research field categorization for each journal was retrieved from the Scimago Journal Database (30-03-23; https://www.scimagojr.com/journalrank.php?out=xls). Note that other adversities (such as interparental violence or bullying) may have been studied in the included publications; these adversities are not presented in our analyses.

**Figure 1.**
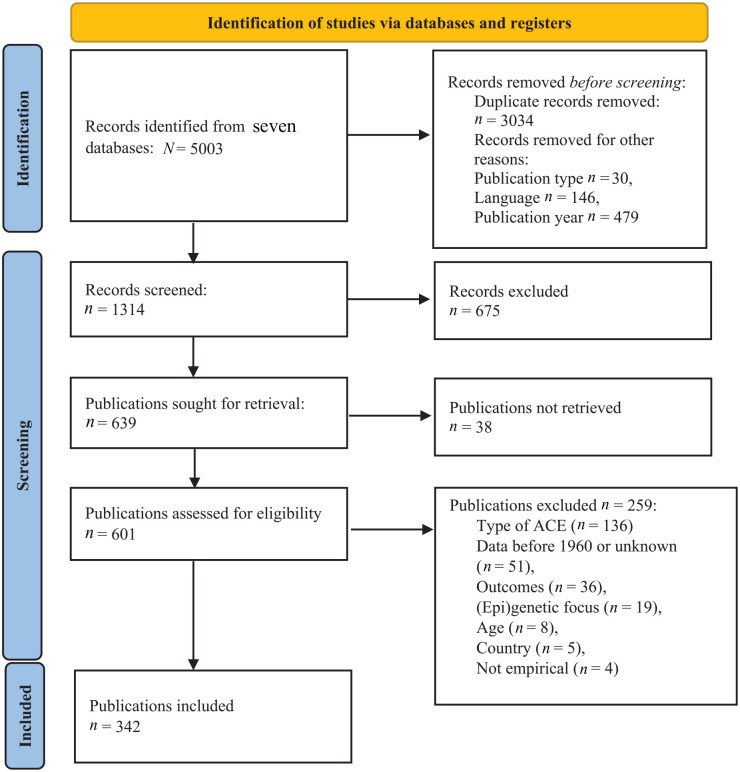
Flow diagram of identification, screening and inclusion process. ACE: adverse childhood experience.

Figure 1 illustrates the article identification, screening and inclusion process in flow chart form [40]. Details on search strategies, inclusion and exclusion criteria, spreadsheet with the included publications and extracted variables, R code and additional figures can be found on the project’s Open Science Framework repository (https://osf.io/xgs4n/) and in the Supplemental material online.

## Results

### Trends and patterns of household dysfunction ACEs research

We first provide an overview using bibliometric analyses to summarize the characteristics of the 342 included articles with regard to publication patterns.

The analysis of the number of publications on household dysfunction ACEs and child outcomes suggests an increasing trend in the number of publications over time (see Supplemental Figure S1). The number of publications retrieved are: *n*=95 for Sweden, *n*=87 for Finland, *n*=79 for Denmark, *n*=72 for Norway, *n*=4 for Iceland and *n*=5 cross-national in the period of interest. Longitudinally, Finland had the greatest number of publications compared with the other countries until 2012. Thereafter, Sweden and Denmark are best represented.

### Household dysfunction ACEs and child outcomes

Household dysfunction ACEs and child outcomes were coded for each publication. Table I provides an overview of the included adversities, descriptions and number of publications regarding the single ACE. In all, one-third of the publications studied two or more household dysfunction ACEs (*n*=112) of interest. For publications that studied only one ACE, divorce/separation was the most common (*n*=97), whereas parental incarceration/criminality was the least common (*n*=9).

**Table I.** Household dysfunction ACEs and descriptions with number of publications.

ACE	Description	Total publications *n*=342 (%)
Multiple and instrument	Any combination of two or more ACEs as described below. Includes validated and non-validated instruments (for example: child household dysfunction items)	*n*=112 (33%)
Divorce/separation	Divorce, separation or parents who are no longer living together	*n*=97 (28%)
Substance abuse	Parental alcohol, drug, tobacco abuse	*n*=34 (10%)
Death	Parental death due to any cause including health shock, accident, suicide, illness	*n*=33 (10%)
Mental health	Parental mental health disorders (for example: depression, anxiety)	*n*=29 (8%)
Physical health	Parental somatic disorders (for example: cancer, multiple sclerosis)	*n*=18 (5%)
ACE as a control only	ACE included as a control (reported in quantitative results)	*n*=10 (3%)
Criminality	Parental incarceration, criminal behaviour	*n*=9 (3%)

ACE: adverse childhood experience.

**Figure 2.**
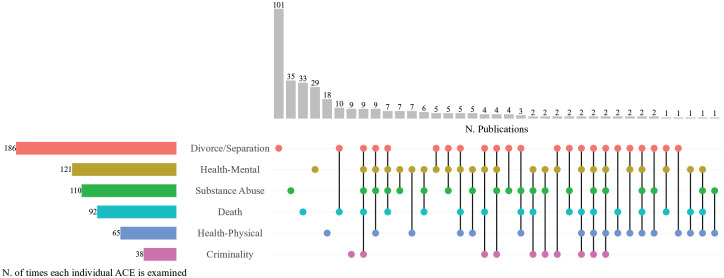
Household dysfunction ACEs (disaggregated) and number of publications. Grey vertical bars represent the number of publications with a specific household dysfunction ACE or combination of these ACEs. Coloured horizontal bars visualize how many times a specific ACE occurs across publications. Dots indicate whether the ACEs of interest are studied individually (single dot) or together with other ACEs of interest (dots connected with black solid lines). ACE: adverse childhood experience.

**Figure 3.**
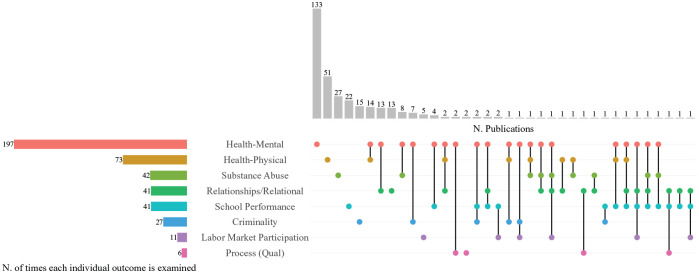
Child outcomes (disaggregated) and number of publications. Grey vertical bars represent the number of publications with a specific child outcome or combination of outcomes. Coloured horizontal bars visualize how many times a specific outcome occurs across publications. Dots indicate whether outcomes are studied individually (single dot) or together with other outcomes (dots connected with black solid lines). qual: qualitative.

As several studies include two or more household dysfunction ACEs and/or child outcomes of interest, we visualize overlaps in Figures 2 and 3 using ‘upset’ plots. Because they depict intersections of ACEs across the literature, the number of appearances is greater than the total number of included publications. The vertical grey bars show the number of times a specific ACE/outcome was studied alone (grey bars over single dot) or together with other ACEs/outcomes (grey bars over multiple dots connected by a solid line). The coloured horizontal bars represent the total number of times a specific ACE/outcome was analysed either alone or together with others. Among household dysfunction ACEs, divorce/separation was the most studied, both alone and in combination (*n*=186) with one or more other adversities. Thereafter, the most commonly occurring ACEs across all the included publications were parental: mental health (*n*=121), substance abuse (*n*=110), death (*n*=92), physical health (*n*=65) and criminality (*n*=38).

Regarding combinations of two or more ACEs, parent death and divorce/separation appeared in the largest number of publications (*n*=10). Only *n*=2 publications investigated all ACEs of interest. Note that the discrepancies between the numbers reported in column 3 of Table I and the vertical grey bars of Figure 2 are owing to ACEs coded as ‘ACE as control’ or ‘Multiple/instrument’.

We also assessed the outcomes of children experiencing the aforementioned ACEs. Mental health was the most common child outcome (*n*=131). Regarding the other child outcomes in the included articles, *n*=51 focused on physical health, *n*=27 on substance abuse, *n*=21 on school, *n*=15 on criminality, *n*=13 on relational factors, *n*=5 on labour market participation and *n*=2 on processes. *n*=71 publications included two or more outcomes of interest in their analyses, and *n*=6 used an instrument. Table II provides an overview of the outcomes included with descriptions and total publications for each outcome.

**Table II.** Child outcomes and descriptions with number of publications.

Outcome of children	Description	Total publications *n*=342
Mental health	Mental health disorders (for example: depression, anxiety), subjective psychological well-being	*n*=131
Multiple	Any combination of two or more outcomes; also includes validated and non-validated measures including and reporting independent results for adversities of interest	*n*=71
Physical health	Somatic disorders (for example: cancer), mortality	*n*=51
Substance abuse	Alcohol abuse, illicit substance abuse, prescription drug abuse, smoking	*n*=27
School	Grades/Grade Point Average, school completion, school dropout, level of educational attainment	*n*=21
Criminality	Incarceration, criminal behaviour	*n*=15
Relational	Relationships within or outside family, including intimate relationships (for example: conflict, closeness, experiences with parenting, loneliness, divorce), social skills	*n*=13
Instrument	Validated and non-validated instruments that include a combination of two or more outcomes (for example: Health-Related Quality of Life)	*n*=6
Labour market	Labour market participation, trajectories, unemployment, NEET status	*n*=5
Process	Mapping experiences or narratives (qualitative only)	*n*=2

NEET: Not in Employment, Education, or Training.

Figure 3 depicts all outcomes related to children who experienced household dysfunction ACEs. Publications with multiple outcomes (*n*=71) are presented in disaggregated form. In all, *n*=197 publications studied mental health outcomes, either alone (*n*=133) or in combination with other outcomes. Physical health outcomes were the second most prevalent, appearing in *n*=73 publications alone (*n*=51) or together with other outcomes. Of publications dealing with multiple child outcomes, the largest number (*n*=14) investigated outcomes related to physical and mental health, followed by mental health and relational aspects (*n*=13). Discrepancies between the numbers reported in column 3 of Table II and the vertical grey bars of Figure 3 are owing to the outcomes coded as ‘Instrument’.

Next, we considered the connections between our household dysfunction ACE(s) of interest and child outcomes, as studied in the included literature. The most common connection was between divorce/separation and child mental health outcomes (*n*=109 publications), followed by parental mental health and child mental health outcomes (*n*=79), parental substance abuse and child mental health outcomes (*n*=59), and parent death and child mental health outcomes (*n*=55). Supplemental Figures S2 and S3 visualize the connections between ACEs and child outcomes.

### Longitudinal patterns

We also observe longitudinal patterns related to household dysfunction ACEs and child outcomes, as coded by the research team (Supplementary Figures S4 and S5). Divorce/separation was the most common adversity of focus until 2015, accounting for *n*=146 (43%) of the published articles. From 2019–2021, parental mental health was the most common, although divorce/separation, parental substance abuse and parental death were also well represented ACEs. Regarding child outcomes, mental health was the most prevalent outcome in the literature in most of the years. Physical health and substance abuse were also common outcomes to assess. In 2016 and 2021–2022, there were increased numbers of publications on school-related outcomes, and in 2016–2018 and 2020 on relational outcomes, although it is unclear whether these represent patterns in the research.

### Methods, data and design

The majority of the publications included (92%; *n*=315) used quantitative methods. This included cohort studies (*n*=199), cross-sectional studies (*n*=108) and other types of quantitative approaches (*n*=8; nested case control studies, mix of cross-sectional and longitudinal design). In total, *n*=24 publications used qualitative approaches. Of these, *n*=19 used interviews and/or focus groups, *n*=4 used other qualitative approaches (open-ended survey responses, written accounts, textual data from digital fora) and *n*=1 used observations. *n*=3 publications used mixed methods, combining qualitative and quantitative approaches (qualitative and descriptive quantitative data, and Q methodology combined with visual images).

Data sources included register data (*n*=142), child self-report (*n*=67), multiple sources (*n*=58, for example, register data combined with cross-sectional self-reported survey data), adult retrospective self-report (*n*=52) and parent self-report (*n*=23). Overall, the most common design was a cohort study using register data (*n*=130).

### Journal categorization

Using the Scimago Journal Database, we categorized the publications into 26 research fields. The largest number of articles appeared in journals related to Medicine (*n*=76). Thereafter, Psychology (*n*=35), Developmental and Educational Psychology (*n*=32) and Psychiatry and Mental Health (*n*=32) journal types were also well represented. In contrast, Paediatrics, Perinatology and Child Health (*n*=14), Law (*n*=14), Arts and Humanities (*n*=13), Social Sciences (*n*=10) and multidisciplinary (*n*=5) journals were represented to a lesser degree. Overall, 78% of the included publications were in health, epidemiology, medicine or psychology journals.

### Protective factors and interventions

The included studies were also coded according to whether they investigated factors described as protective, or which facilitated child resilience after exposure to household dysfunction ACEs (see Supplemental Table SI). These were further coded based on the category of protective factors (individual, family, contextual or multiple). Of all the included studies, *n*=36 (11%) investigated protective factors or resilience and *n*=7 of these investigated associations between protective factors and outcomes, independently of household dysfunction ACEs. Only *n*=2 included measures on all three categories, assessing individual, familial and contextual resilience factors [17,47]. *n*=14 publications included measures from two categories and *n*=20 from only one. In total, *n*=25 articles included family factors (such as quality of the relationship with parents, parental support, parent education). Contextual factors (such as social support, school support, neighbourhood deprivation) were included in *n*=18 articles, of which the majority investigated social support measured by only a few items in a questionnaire. A total of *n*=11 studies included individual factors (such as school achievement, stress tolerance, sense of coherence, prosocial behaviour, satisfaction with living arrangements). Mirroring the overall body of literature on household dysfunction ACEs, the majority (*n*=32) of these publications on protective factors were quantitative, whereas *n*=4 were qualitative.

In addition to studies investigating protective factors, *n*=10 publications have explored the role of specific interventions, such as formal health interventions or informal online self-help groups. Overall, these were found to have positive outcomes regarding supporting young people (and sometimes their families) dealing with specific household dysfunction ACEs. However, none of these studies used randomized controlled trials (RCTs).

## Discussion

In this bibliometric analysis of 342 articles, we mapped the research on household dysfunction ACEs across the five Nordic countries, uncovering a substantial and growing body of literature. The comprehensive nature of our analysis provides a basis for understanding overall trends and suggesting directions for future inquiry based on gaps in the extant literature.

The most frequently studied household dysfunction ACEs were parental divorce/separation and mental health, whereas the least studied were parental physical health and incarceration/criminality. Although this finding on incarceration may be due to the relatively low prevalence of criminality in the Nordic population or challenges in data acquisition, it limits evidence of the impact of these ACEs on children. Children who have experienced parental incarceration can be considered a particularly vulnerable group, especially in the Nordic context, where incarceration is relatively rare [48]. Information on the specific challenges and outcomes related to parental incarceration can help ensure sufficient support for these children. The largest share of the articles (33%) studied the ACEs jointly, either by including combinations of adversities or instruments in a cumulative approach. This trend remained stable throughout the selected period. While this approach can highlight the cumulative effects of childhood adversities, which often are interrelated [18], research focusing on specific household dysfunction ACEs can provide information suited to addressing specific and targeted interventions for children experiencing these ACEs.

The most studied child outcomes are related to the mental and physical health of those who experienced household dysfunction ACEs during childhood or adolescence. This corresponds to international reviews and bibliometric analyses that focus on a wider range of adversities, beyond those related to parents. These overwhelmingly include publications on mental and physical health outcomes, but also substance abuse [29]. Conversely, there are few publications on outcomes related to criminality, processes or social relationships in our included literature. We also observe fewer publications on functional outcomes, such as education or labour market participation, although there has been an increased focus, especially on educational outcomes, in recent years. This is promising, given the importance of formal educational qualifications for participating in working life in the Nordic countries [49], and thus the salience of schools as an arena for reducing exclusion.

Reflecting on our findings in conjunction with earlier reviews, it is evident that employing household dysfunction ACEs as a lens offers valuable insights into the transmission of adversities across generations. By extending the scope of investigation beyond exclusively health outcomes to include broader social and functional domains, we can develop a more comprehensive framework to examine youth exclusion, a pressing concern in Nordic countries and a target of various UN Sustainable Development Goals. Nevertheless, integrating socioeconomic considerations remains critical for fully understanding these dynamics (as highlighted by Taylor-Robinson et al. [50] and Pitkänen et al. [51]).

Notably, the role of protective factors and resilience are relatively unexplored in our literature, being included in only 11% of the publications. Furthermore, few publications studied interventions (3%) and none used an RCT design. This finding also coincides with previous international reviews, which underscore that research often investigates the downstream effects of childhood adversities rather than the causes or potential preventive factors or interventions [7]. It also supports a recent international systematic review, which included 37 studies on protective factors in children of parents with mental illness, finding that the research field is scarce [37], even for this well-studied adversity. It could, therefore, be beneficial for the ACEs literature to move toward intervention, treatment and prevention-based studies. This approach would require a more structured causal inference design, which is currently lacking in the literature, in part because of the ethical difficulties of defining treatment and control groups with ACEs. Such a shift would result in more national and local rather than cross-national studies, with smaller sample sizes and more homogenous populations with respect to the current state of the art.

A related strategy would be to deepen the understanding of the role of protective factors and development of resilience. Of the included publications which investigated protective factors, only two examined measures related to all three coded categories (individual, family, contextual). Most of our publications dealing with protective factors focused exclusively on the family, in the form of parental support and the quality of the relationship with parents. This contrasts with recent research suggesting that resilience-promoting interventions should take a holistic view, targeting multiple areas of a person’s life [52]. It is important to increase the focus on protective factors related to the broader social context in the context of household dysfunction ACEs. The studies that included contextual factors mainly focused on brief measures of social support. The literature would benefit from a broader and more inclusive investigation of contextual factors that can be bolstered, for instance, through schools, public institutions and leisure activities. Furthermore, an international meta-analysis suggests that individuals who experienced an ACE were 63% less likely to exhibit strong resilience compared with individuals who had not experienced an ACE [53]. This underscores a need for tailored, effective interventions to support young people and their families and corresponding studies on their implementation and effects.

Methodologically, most (92%) of the studies used quantitative methods, with data sources from public registers or self-reports, and retrospective designs. Although these methods have been instrumental in quantifying the association between ACEs and various health outcomes, our analysis reveals a critical need for diversifying methodological approaches to capture the full complexity of these experiences. Future studies could exploit the potential to combine the rich register data available in the Nordic countries with surveys or qualitative methods. Further, because it may be difficult to use register data to operationalize certain domains contributing to resilience (such as family functioning or self-esteem), self-reported surveys could provide this information. Such approaches can allow the mapping of different trajectories longitudinally and prospectively and identify critical junctures for intervention and support. This approach could enhance our understanding of the long-term effects of adversities, and it is also aligned with the need for evidence-based policy-making that can address the intergenerational transmission of these experiences. The underutilization (8%) of qualitative and mixed-methods studies in our literature review highlights a broader issue within the field. These methodologies are crucial for analysing the subjective experiences of those affected by household dysfunction ACEs, the complex interplay of factors and the underlying mechanisms of impact, offering perspectives that quantitative data alone cannot provide. Further, understanding mechanisms is essential for developing more effective interventions, as asserted by Lacey and Minnis [20]. We also note that the included qualitative studies utilize ‘traditional’ ethnographic approaches, such as interviews or focus groups, almost exclusively. These approaches could be complemented with analyses of existing organic textual data from sources such as social media or online forums, which might reduce recall biases, although they introduce new methodological challenges [54]. Finally, given that our review included few cross-national studies, this approach might also provide new insights, although dissimilarities across available data pose an ever-present challenge.

Most articles (78%) were published in health-related journals, whereas few were published in fields related to social sciences or humanities. This is partially in line with findings from international bibliometric analyses using researcher-coded journal research fields, which also included the majority of publications from health, but also had 20% of articles from ‘multidisciplinary’ journals [29]. Given the ACE term’s psychology origins [1], the health focus is not entirely surprising. However, we anticipated improving this disciplinary bias by explicitly searching for specific adversities. This suggests that our knowledge of household dysfunction ACEs and child outcomes stems primarily from a health perspective, which is in line with the primacy of health related adversities and outcomes in our included literature as well as in previous international reviews. In contrast, approaches based on sociological, public administration or economic traditions remain relatively unexplored. Integrative, multidisciplinary approaches could therefore further expand the research field, possibly by broadening the focus beyond health-related adversities and outcomes. A multidisciplinary approach could also be beneficial for studying resilience, incorporating new perspectives to complement existing psychology and public health dominance.

### Limitations

This study has some limitations. We included only peer-reviewed articles in English and excluded those using Nordic languages and grey literature such as reports. Thus, we may have missed the rich and detailed findings included in the grey literature and insights presented in other languages. However, since no members of the research team understood Icelandic or Finnish languages, the inclusion of grey literature would likely have biased our included publications towards the other three countries. In addition, our search criteria included variations on the word ‘parent’ rather than specifying mother and/or father, and the review may therefore have missed literature that included only these gender-specific terms. However, our search in article abstracts in addition to title and keywords may improve this omission to some extent. Our search also did not use the term ‘household dysfunction’; however, we expect that searching for specific adversities within this category would balance this omission. A second limitation in our conceptualization of household dysfunction is the exclusion of inter-partner violence, though research suggests it warrants its own category within the ACE framework owing to its distinct developmental impact pathways (see Holt et al. [55]).

While our article assumes that the included adversities typically decrease parents’ capacity to care for their children, we acknowledge that individual cases may differ. For instance, some exposure to an adversity might increase parental investments, such as a parent reprioritizing family time after a separation. However, research suggests (cf. Kalil et al. [56] and Menaghan et al. [57]) that ACEs generally reduce the emotional and practical resources that parents invest in their children.

In addition, the coding of articles is highly dependent on the individual coders. Despite efforts like written criteria for coding categories developed as a team, testing, group coding, frequent meetings, and discussions in cases of uncertainty, there may be variation across how individual team members coded articles, and it is possible that a different research team would have returned slightly different results. This is particularly relevant regarding protective factors, which could be challenging to identify and were discussed within the team. However, our results on protective factors coincide with previous international research on ACEs more broadly [7,37], strengthening their validity. Finally, although bibliometric analyses provide a comprehensive, quantitative overview of the extant literature on parental ACEs, a detailed analysis of the contents of these hundreds of publications is beyond the scope of this article. Computational text analyses or qualitative analyses of subsamples of the included literature could be the next step in this direction. Readers particularly interested in the individual findings for each included article may check the ‘abstract’ column in the Supplemental data extraction file.

## Conclusion

Overall, our analyses have provided a comprehensive overview of household dysfunction ACEs in the Nordic countries while also facilitating the identification of gaps in the literature. In line with previous international reviews, both household dysfunction ACEs and child outcomes are dominated by a mental health perspective. We identified few publications addressing protective factors for children. Research on household dysfunction ACEs could benefit from a methodological shift toward more integrated approaches using multidisciplinary perspectives to better understand the complex developmental processes involved. Embracing a broader range of research methods and exploiting the unique data resources available in Nordic countries, this field can uncover new insights on adversity, childhood outcomes and resilience, and inform evidence-based interventions for children exposed to these ACEs.

## Supplemental Material

sj-docx-1-sjp-10.1177_14034948251336851 – Supplemental material for Household dysfunction and child outcomes in the Nordic countries: A bibliometric analysis Supplemental material, sj-docx-1-sjp-10.1177_14034948251336851 for Household dysfunction and child outcomes in the Nordic countries: A bibliometric analysis by Rebecca L. Radlick, Alice Milivinti, Håvard T. Rydland, Ingrid R. Lundeberg and Kristin G. Askeland in Scandinavian Journal of Public Health

sj-docx-2-sjp-10.1177_14034948251336851 – Supplemental material for Household dysfunction and child outcomes in the Nordic countries: A bibliometric analysis Supplemental material, sj-docx-2-sjp-10.1177_14034948251336851 for Household dysfunction and child outcomes in the Nordic countries: A bibliometric analysis by Rebecca L. Radlick, Alice Milivinti, Håvard T. Rydland, Ingrid R. Lundeberg and Kristin G. Askeland in Scandinavian Journal of Public Health

sj-docx-3-sjp-10.1177_14034948251336851 – Supplemental material for Household dysfunction and child outcomes in the Nordic countries: A bibliometric analysis Supplemental material, sj-docx-3-sjp-10.1177_14034948251336851 for Household dysfunction and child outcomes in the Nordic countries: A bibliometric analysis by Rebecca L. Radlick, Alice Milivinti, Håvard T. Rydland, Ingrid R. Lundeberg and Kristin G. Askeland in Scandinavian Journal of Public Health
