# Supplementary material for: Household dysfunction and child outcomes in the Nordic countries: A bibliometric analysis
Source: Scand J Public Health. 2025 May 24;53(6):658–69. doi: 10.1177/14034948251336851 (PMC12374006; doi:10.1177/14034948251336851)
Supplement: sj-docx-1-sjp-10.1177_14034948251336851 – Supplemental material for Household dysfunction and child outcomes in the Nordic countries: A bibliometric analysis [file sj-docx-1-sjp-10.1177_14034948251336851.docx]

**Supplemental Material: Figures & Tables**

**Supplemental Figures**


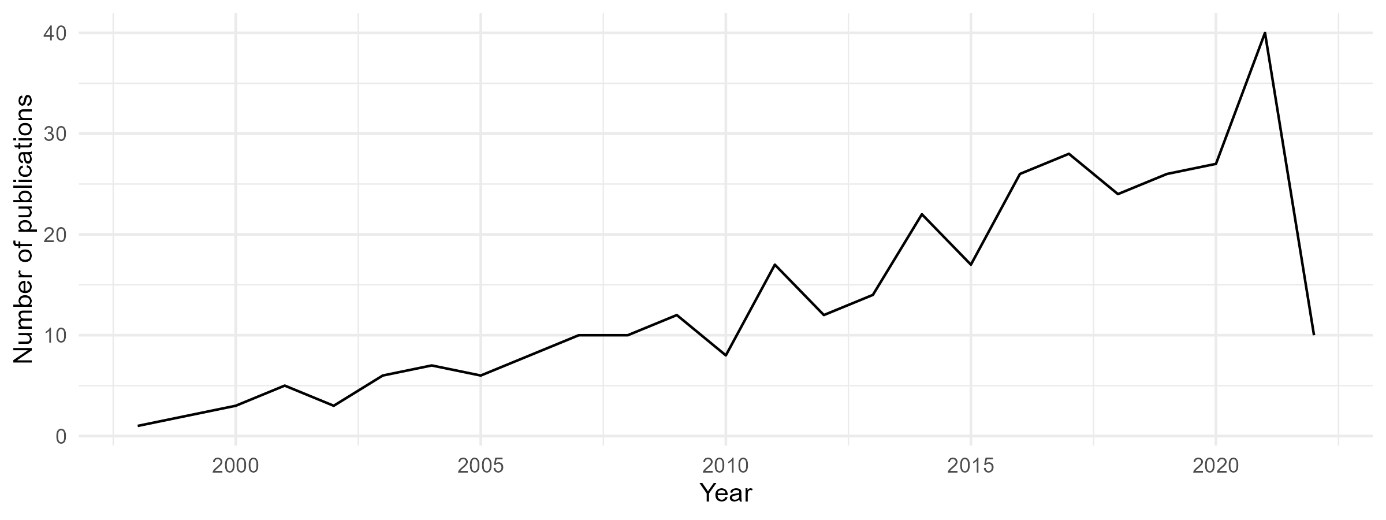


**Figure S1.** *Number of publications from January 1998 to April 2022*

*Our database search was conducted in April of 2022, hence the drop from 2021, due to not having one full year of data


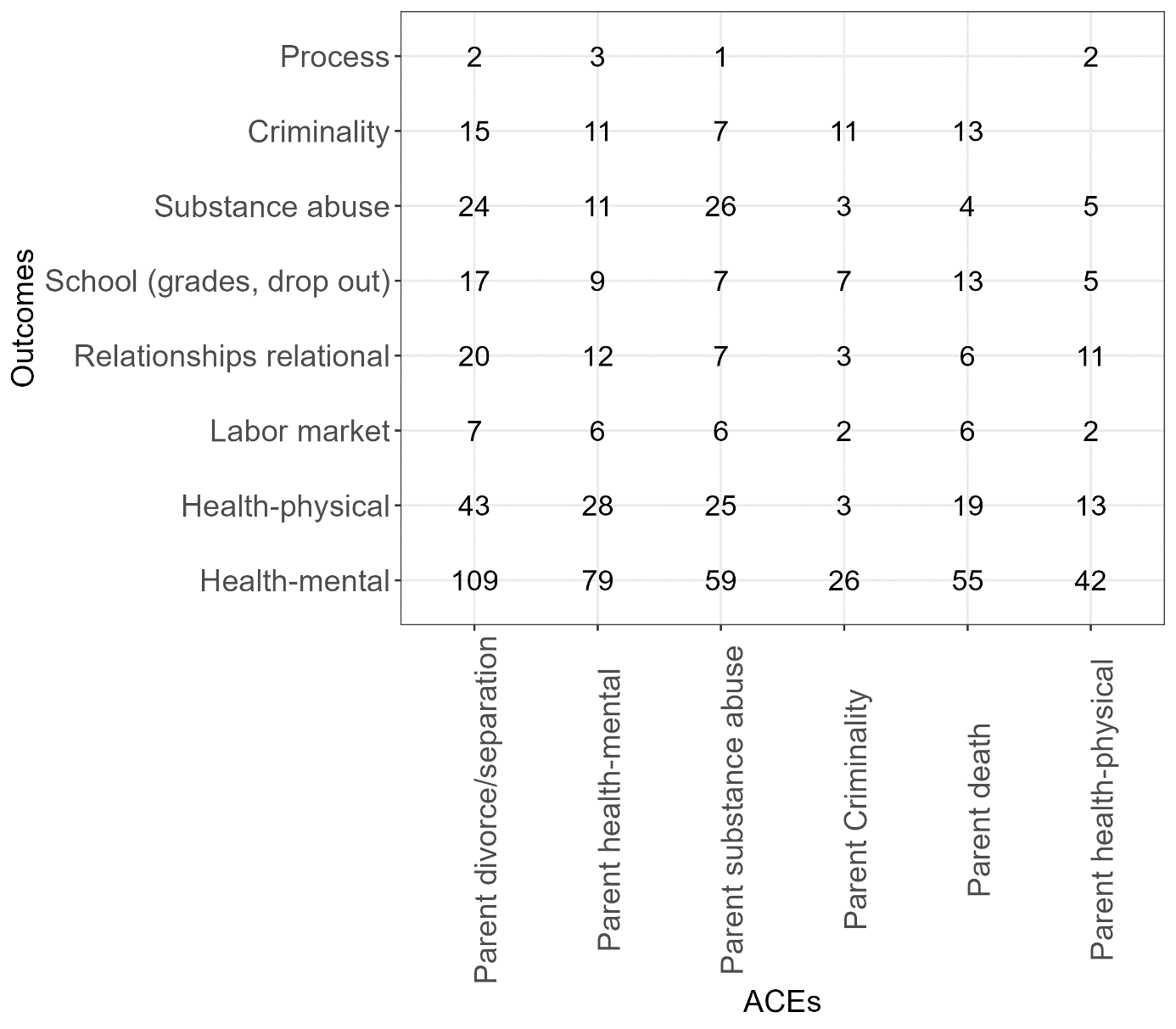


**Figure S2.** *Child outcomes vs household dysfunction ACEs combinations found in the literature*


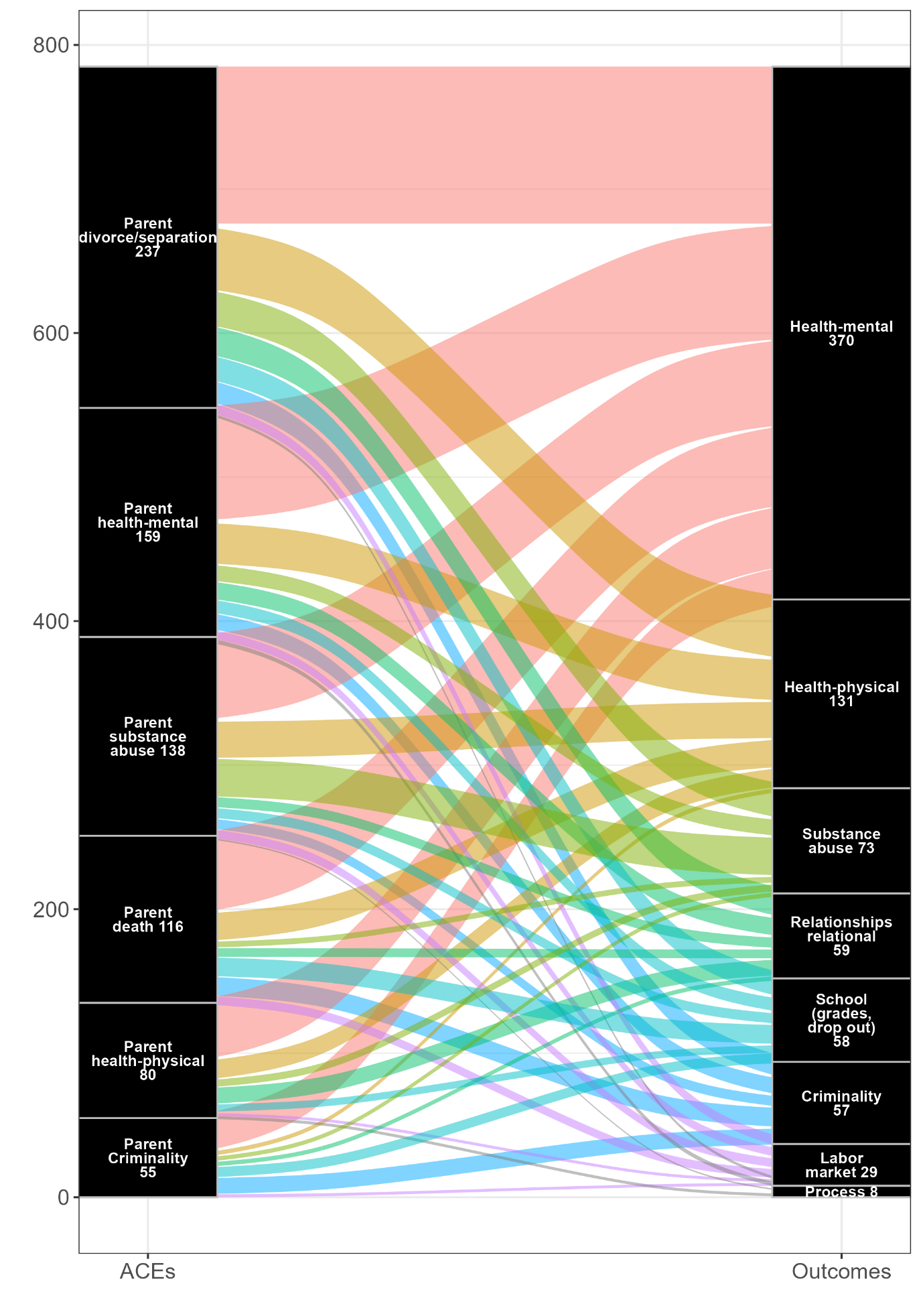
**Figure S3.** *Household dysfunction ACEs and child outcomes found in the literature*

***** Publications with multiple ACEs or outcomes are disaggregated and depicted individually.


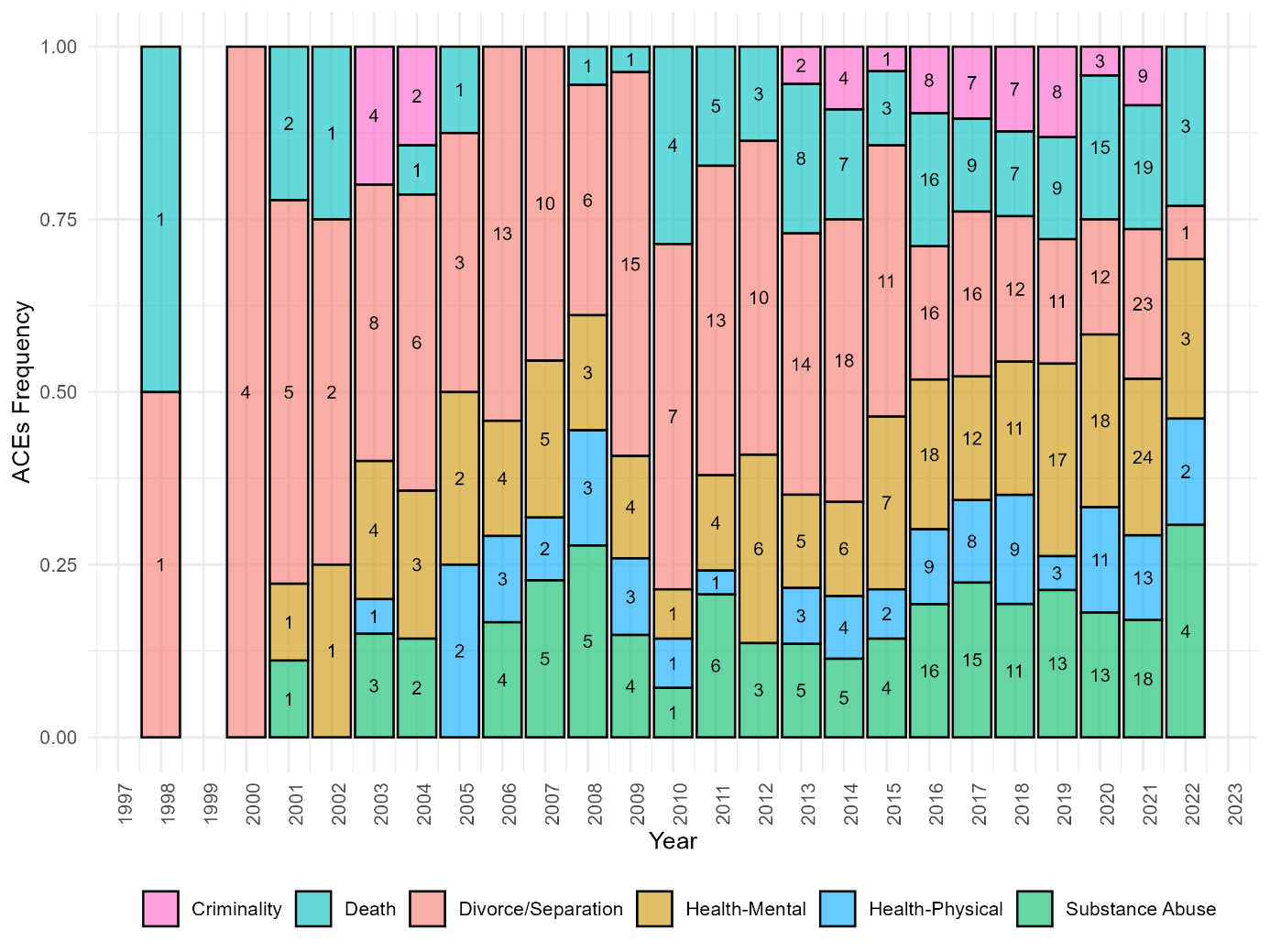


**Figure S4.** *Household dysfunction ACEs frequencies in publications by year from 1998 to 2022. Absolute numbers are reported within the bars.*


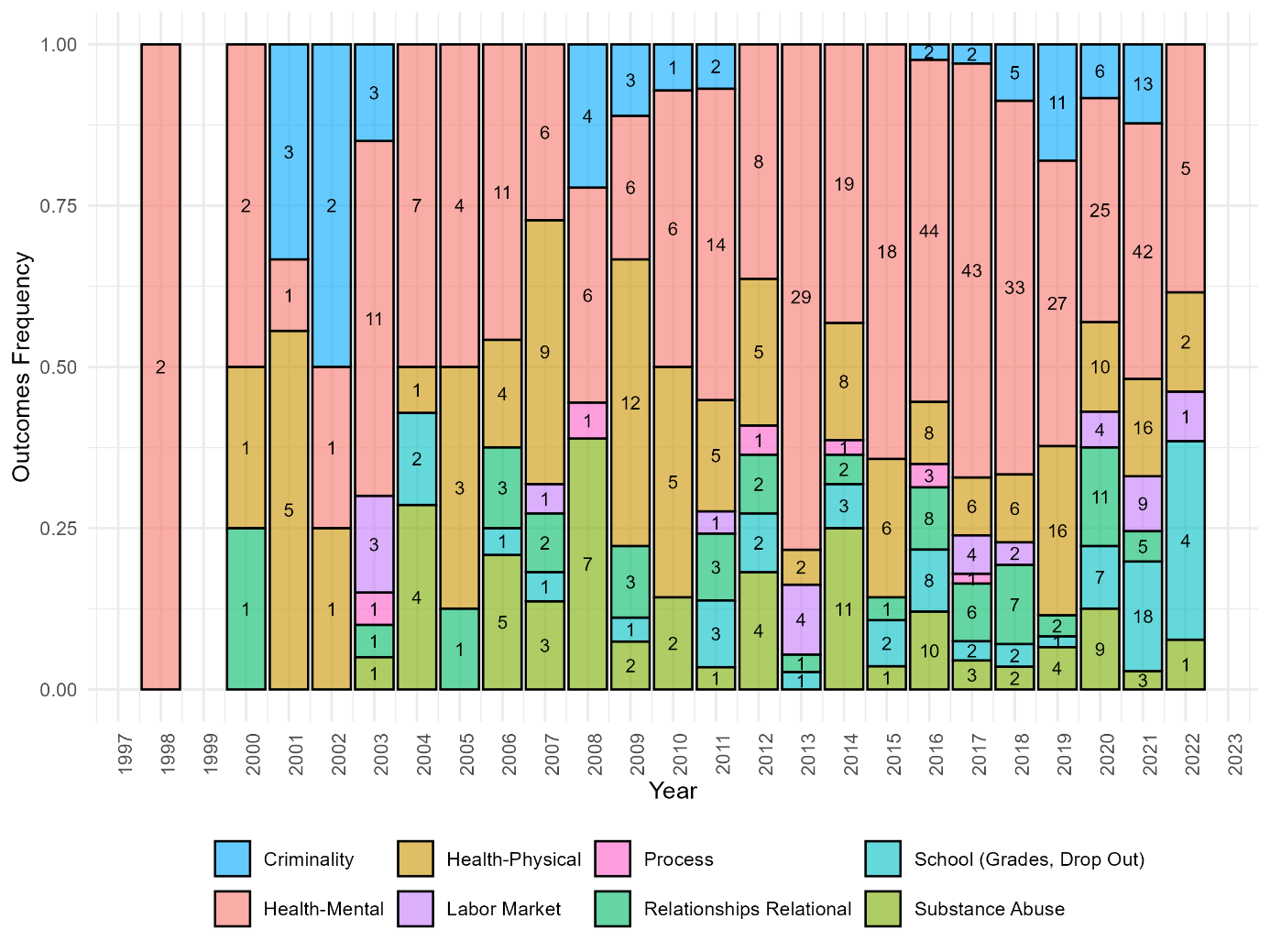


**Figure S5.** *Child outcomes frequencies in publications by year from 1998 to 2022. Absolute numbers are reported within the bars.*

**Table S1**. *Protective factors, examples, and number of publications*

| **Resilience** | **Examples** | **Number of publications** |
| --- | --- | --- |
| Individual factors | School achievement, stress tolerance, physical fitness, sense of coherence, stress, satisfaction with living arrangements, prosocial behavior | 5 |
| Family factors | Family functioning, parent education, socioeconomic status (SES) | 9 |
| Contextual factors | Social support, school-related (student-teacher relationship, classroom community), neighborhood deprivation/local SES | 6 |
| Individual & family | Combination of individual and family level factors | 4 |
| Family & context | Combination of family and contextual level factors | 10 |
| All 3 (individual, family, context) | Combination of individual, family, and contextual level factors | 2 |
