# Supplementary material for: Household dysfunction and child outcomes in the Nordic countries: A bibliometric analysis
Source: Scand J Public Health. 2025 May 24;53(6):658–69. doi: 10.1177/14034948251336851 (PMC12374006; doi:10.1177/14034948251336851)
Supplement: sj-docx-2-sjp-10.1177_14034948251336851 – Supplemental material for Household dysfunction and child outcomes in the Nordic countries: A bibliometric analysis [file sj-docx-2-sjp-10.1177_14034948251336851.docx]

**Household dysfunction ACEs:**

**Inclusion/exclusion criteria and search terms**

**Inclusion and exclusion criteria**

***Inclusion criteria***

- Peer reviewed journal article
- Original, empirical research
- English
- Includes at least one of the five Nordic countries (Norway, Sweden, Finland, Denmark, Iceland)
- Use qualitative, quantitative, or mixed-methods approaches
- Outcomes related to the child (either still as a child or when they are older; no age range limit)
- Adversity started/occurred before the child turned 18
- Publishing date from 1998 or later (Post search criterion)
- ACEs occurred after 1960 (Post search criterion)
- Publication can include parental criminality (in addition to incarceration) (Post search criterion)
- If multiple ACEs are studied in combination (for example, as part of an instrument/screening tool), results for our individual ACEs of interest are reported (for example quantitatively in a table) (Post search criterion)

***Exclusion criteria***

- Not peer reviewed journal article (ie. grey literature, books, etc.)
- Protocol with no results presented or purely theoretical
- Not written in English
- Does not include one of the five Nordic countries
- Includes at least one of the five Nordic countries, but also non-Nordic countries (for example in multiple country or cross-national comparative analyses)
- A review article (in this case we will likely cite it, but not include in the analyses)
- Outcomes not focused on the child
- Adversity started/occurred after the child turned 18
- Publication date prior to 1998 (ACE study) (Post search criterion)
- ACEs occurred prior to 1960 (Post search criterion)
- Results for our individual ACEs of interest are not reported when multiple ACEs are studied in combination (for example, as part of an instrument/screening tool) (Post search criterion)

**Databases for search**

Web of Science

PsycINFO

Medline

Embase
ERIC

CINAHL

Scopus

**Proposed search terms:**

The original ACE study covers domains, encompassing interrelated categories: abuse (emotional, physical, sexual), neglect (psychological, physical), and household dysfunction (substance abuse, illness/death, parental separation/divorce, and household legal involvement). We focus on adversities linked to parents (“parental ACEs”) which overlap with household dysfunction. We conducted two searches for each database, combining results. The first search focused on ACE literature in the Nordic countries explicitly. This entailed Boolean search string encompassing the empirical context of 5 Nordic countries (“0” below) and variations on the term “adverse childhood experiences” (adverse childhood events, childhood adversities) (“1” below).

The second focused on specific adversities related to the parents. Search terms included the Nordic countries (“0” below), variations on the word child (adolescent, youth), since we are interested in various child outcomes (rather than only on parental ACEs), as well as variations on specific types of family adversities related to parental incarceration, substance abuse, divorce, and parental illness/health shocks (“2” below).

This approach enabled us to identify publications which were not explicitly grounded in the “Adverse Childhood Experiences” literature, aiming to also include publications from a broad range of research disciplines.

**Search terms**

1. *The empirical context (Nordic countries)*

Norway* OR Norwegian OR Sweden* OR Swedish OR Denmark* OR Danish OR Finland* OR Finnish OR Iceland* OR Icelandic

1. *ACE literature broadly*

Norway* OR Norwegian OR Sweden* OR Swedish OR Denmark* OR Danish OR Finland* OR Finnish OR Iceland* OR Icelandic

AND

“adverse childhood experience*”

“adverse childhood event*”

“childhood adversit*”

1. *ACEs (specific adversities)*

Norway* OR Norwegian OR Sweden* OR Swedish OR Denmark* OR Danish OR Finland* OR Finnish OR Iceland* OR Icelandic

AND

Child* OR Youth* OR Adolescen*

AND

**Parental addiction**

“parent* addict*”

Parent* substance use

“parent* substance abuse”

“parent* alcohol use”

“parent* alcohol abuse”

OR

**Parent illness/health shocks**

“parent* ill*”

“Parent* physical ill*”

“parent* mental* ill*”

“parent* chronic* ill*”

“parent* psych* ill*”

“parent* death”

“parent* health shock*”

OR

**Divorce**

“parent* separate*”

“separate* parent*”

Divorce*

“marital dissolution”

OR

**Incarceration**

“parent* incarcera*”

“parent* imprison*”
