# Supplementary material for: Household dysfunction and child outcomes in the Nordic countries: A bibliometric analysis
Source: Scand J Public Health. 2025 May 24;53(6):658–69. doi: 10.1177/14034948251336851 (PMC12374006; doi:10.1177/14034948251336851)
Supplement: sj-docx-3-sjp-10.1177_14034948251336851 – Supplemental material for Household dysfunction and child outcomes in the Nordic countries: A bibliometric analysis [file sj-docx-3-sjp-10.1177_14034948251336851.docx]

**Reference List: Included Articles**

A. A. Morgan, J. A. Arditti, S. Dennison and S. Frederiksen. (2021). Against the odds: A structural equation analysis of family resilience processes during paternal incarceration. International Journal of Environmental Research and Public Health, 18(21), 11592

A. Alvariza, M. Lovgren, T. Bylund-Grenklo, P. Hakola, C. J. Furst and U. Kreicbergs. (2017). How to support teenagers who are losing a parent to cancer: Bereaved young adults' advice to healthcare professionals-A nationwide survey. Palliative & supportive care, 15(3), 313-319

A. Astrup, C. B. Pedersen, P. L. H. Mok, M. J. Carr and R. T. Webb. (2017). Self-harm risk between adolescence and midlife in people who experienced separation from one or both parents during childhood. Journal of affective disorders, 208, 582-589

A. Biehl, R. Hovengen, E. K. Groholt, J. Hjelmesaeth, B. H. Strand and H. E. Meyer. (2014). Parental marital status and childhood overweight and obesity in Norway: A nationally representative cross-sectional study. BMJ Open, 4(6), e004502

A. Carlsund, U. Eriksson and E. Sellstrom. (2013). Shared physical custody after family split-up: implications for health and well-being in Swedish schoolchildren. Acta paediatrica (Oslo, Norway : 1992), 102(3), 318-23

A. Faugli, E. Kufas, M. Haukland, E. K. Kallander, T. Ruud and B. M. Weimand. (2021). 'I have cried a lot': a qualitative study on children experiencing severe parental illness. Scandinavian journal of caring sciences, 35(4), 1196-1206

A. G. Franzen and L. Gottzen. (2021). Childhood adversities and individual responsibility as explanations for criminality in incarcerated young men's narratives. Deviant Behavior, No-Specified

A. Hjern, M. Bergstrom, E. Fransson and S. Kjaer Urhoj. (2021). Living arrangements after parental separation have minimal impact on mental health at age 7 years. Acta paediatrica (Oslo, Norway : 1992), 110(9), 2586-2593

A. Hjern, S. K. Urhoj, E. Fransson and M. Bergstrom. (2021). Mental Health in Schoolchildren in Joint Physical Custody: A Longitudinal Study. Children (Basel, Switzerland), 8(6)

A. Kalil, M. Mogstad, M. Rege and M. Votruba. (2011). Divorced Fathers' Proximity and Children's Long-Run Outcomes: Evidence From Norwegian Registry Data. Demography, 48(3), 1005-1027

A. Kallstrom, K. Hellfeldt and P.-A. Nylander. (2019). Parental imprisonment, child victimization and adult problems. European Journal of Criminology, 16(6), 671-688

A. L. Kristjansson, I. D. Sigfusdottir, J. P. Allegrante and A. R. Helgason. (2009). Parental divorce and adolescent cigarette smoking and alcohol use: Assessing the importance of family conflict. Acta Paediatrica, International Journal of Paediatrics, 98(3), 537-542

A. M. Koponen, N. M. Nissinen, M. Gissler, I. Autti-Ramo, T. Sarkola and H. Kahila. (2020). Prenatal substance exposure, adverse childhood experiences and diagnosed mental and behavioral disorders - A longitudinal register-based matched cohort study in Finland. SSM - Population Health, 11, 100625

A. M. Trauelsen, S. Bendall, J. E. Jansen, H.-G. L. Nielsen, M. B. Pedersen, C. H. Trier, U. H. Haahr and E. Simonsen. (2015). Childhood adversity specificity and dose-response effect in non-affective first-episode psychosis. Schizophrenia Research, 165(1), 52-59

A. M. Undheim and A. M. Sund. (2005). School factors and the emergence of depressive symptoms among young Norwegian adolescents. European Child and Adolescent Psychiatry, Supplement, 14(8), 446-453

A. Nevriana, M. Pierce, C. Dalman, S. Wicks, M. Hasselberg, H. Hope, K. M. Abel and K. Kosidou. (2020). Association between maternal and paternal mental illness and risk of injuries in children and adolescents: nationwide register based cohort study in Sweden. BMJ (Clinical research ed.), 369, m853

A. Ranning, T. Laursen, E. Agerbo, A. Thorup, C. Hjorthoj, J. R. M. Jepsen and M. Nordentoft. (2018). School performance from primary education in the adolescent offspring of parents with schizophrenia and bipolar disorder- a national, register-based study. Psychological medicine, 48(12), 1993-2000

A. Reneflot. (2011). Childhood Family Structure and Reproductive Behaviour in Early Adulthood in Norway. EUROPEAN SOCIOLOGICAL REVIEW, 27(1), 56-69

A. S. Dissing, N. Dich, A.-M. N. Andersen, R. Lund and N. H. Rod. (2017). Parental break-ups and stress: roles of age & family structure in 44 509 pre-adolescent children. European journal of public health, 27(5), 829-834

A. S. T. Anker and L. H. Andersen. (2021). Does the intergenerational transmission of crime depend on family complexity? JOURNAL OF MARRIAGE AND FAMILY, 83(5), 1268-1286

A. S. T. Anker. (2021). Educational Consequences of Paternal Incarceration: Evidence from a Danish Policy Reform. JOURNAL OF QUANTITATIVE CRIMINOLOGY

A. Salonsalmi, O. Pietilainen, E. Lahelma and O. Rahkonen. (2019). Childhood adversities, parental education and disability retirement among Finnish municipal employees. PLoS ONE, 14(7), e0219421

A. Salonsalmi, O. Pietilainen, E. Lahelma, O. Rahkonen and T. Lallukka. (2021). Contributions of childhood adversities to chronic pain among mid-life employees. Scandinavian journal of public health, 1403494820981509

A. Sauvola, J. Miettunen, M. R. Jarvelin and P. Rasanen. (2001). An examination between single-parent family background and drunk driving in adulthood: Findings from the Northern Finland 1966 Birth Cohort. Alcoholism: Clinical and Experimental Research, 25(2), 206-209

A. Sauvola, O. Koskinen, J. Jokelainen, H. Hakko, M. R. Jarvelin and P. Rasanen. (2002). Family type and criminal behaviour of male offspring: The Northern Finland 1966 birth cohort study. International Journal of Social Psychiatry, 48(2), 115-121

A. Sauvola, P. K. Rasanen, M. I. Joukamaa, J. Jokelainen, M. R. Jarvelin and M. K. Isohanni. (2001). Mortality of young adults in relation to single-parent family background - A prospective study of the Northern Finland 1966 birth cohort. EUROPEAN JOURNAL OF PUBLIC HEALTH, 11(3), 284-286

A. Sauvola, T. Makikyro, J. Jokelainen, M. Joukamaa, M. R. Jarvelin and M. Isohanni. (2000). Single-parent family background and physical illness in adulthood: a follow-up study of the Northern Finland 1966 Birth Cohort. SCANDINAVIAN JOURNAL OF PUBLIC HEALTH, 28(2), 95-101

A. Sepa, A. Frodi and J. Ludvigsson. (2005). Mothers' experiences of serious life events increase the risk of diabetes-related autoimmunity in their children. Diabetes Care, 28(10), 2394-2399

A. Sourander and L. Helstelä. (2005). Childhood predictors of externalizing and internalizing problems in adolescence: a prospective follow-up study from age 8 to 16. European Child & Adolescent Psychiatry, 14(8), 415-423

A. Taanila, A. Yliherva, M. Kaakinen, I. Moilanen and H. Ebeling. (2011). An epidemiological study on Finnish school-aged children with learning difficulties and behavioural problems. INTERNATIONAL JOURNAL OF CIRCUMPOLAR HEALTH, 70(1), 59-71

A. Varinen, E. Kosunen, K. Mattila, T. Koskela and M. Sumanen. (2017). The relationship between childhood adversities and fibromyalgia in the general population. Journal of Psychosomatic Research, 99, 137-142

A. Vikat, A. Rimpela, E. Kosunen and M. Rimpela. (2002). Sociodemographic differences in the occurrence of teenage pregnancies in Finland in 1987-1998: a follow up study. JOURNAL OF EPIDEMIOLOGY AND COMMUNITY HEALTH, 56(9), 659-668

A. von Borczyskowski, F. Lindblad, B. Vinnerljung, R. Reintjes and A. Hjern. (2011). Familial factors and suicide: an adoption study in a Swedish National Cohort. Psychological medicine, 41(4), 749-758

B. H. Esbjorn, E. Levin, M. Hoeyer and J. Dyrborg. (2008). Child and Adolescent Psychiatry and Family Status A Nationwide Register-based Study. EUROPEAN CHILD & ADOLESCENT PSYCHIATRY, 17(8), 484-490

B. K. Lyngsoe, T. Munk-Olsen, C. H. Vestergaard, D. Rytter, K. S. Christensen and B. H. Bech. (2021). Maternal depression and childhood injury risk: A population-based cohort study in Denmark. Brain and Behavior, 11(3), e02029

B. Kennedy, U. Valdimarsdottir, K. Sundstrom, P. Sparen, M. Lambe, K. Fall and F. Fang. (2014). Loss of a parent and the risk of cancer in early life: A nationwide cohort study. Cancer Causes & Control, 25(4), 499-506

B. L. Hoeg, C. Johansen, J. Christensen, K. Frederiksen, S. O. Dalton, A. Dyregrov, P. Boge, A. Dencker and P. E. Bidstrup. (2018). Early parental loss and intimate relationships in adulthood: A nationwide study. Developmental psychology, 54(5), 963-974

B. L. Hoeg, C. Johansen, J. Christensen, K. Frederiksen, S. O. Dalton, P. Boge, A. Dencker, A. Dyregrov and P. E. Bidstrup. (2019). Does losing a parent early influence the education you obtain? A nationwide cohort study in Denmark. Journal of public health (Oxford, England), 41(2), 296-304

B. L. Hoeg, J. Christensen, L. Banko, K. Frederiksen, C. W. Appel, S. O. Dalton, A. Dyregrov, M.-B. Guldin, S. E. Jorgensen, M. Lytje, P. Boge and P. E. Bidstrup. (2021). Psychotropic medication among children who experience parental death to cancer. European Child & Adolescent Psychiatry, No-Specified

B. Larsson and A. M. Sund. (2007). Emotional/behavioural, social correlates and one-year predictors of frequent pains among early adolescents: Influences of pain characteristics. EUROPEAN JOURNAL OF PAIN, 11(1), 57-65

B. Larsson, J. F. Sigurdson and A. M. Sund. (2018). Long-term follow-up of a community sample of adolescents with frequent headaches. The journal of headache and pain, 19(1), 79

B. Laugesen, C. Mohr-Jensen, S. K. Boldsen, R. Jorgensen, E. E. Sorensen, M. Gronkjaer, P. Rasmussen and M. B. Lauritsen. (2018). Attention Deficit Hyperactivity Disorder in Childhood: Healthcare Use in a Danish Birth Cohort during the First 12 Years of Life. Journal of Pediatrics, 197, 233-240

B. Reigstad and S. Kvernmo. (2016). Concurrent adversities among adolescents with conduct problems: The NAAHS study. Social Psychiatry and Psychiatric Epidemiology: The International Journal for Research in Social and Genetic Epidemiology and Mental Health Services, 51(10), 1429-1438

B. Reigstad and S. Kvernmo. (2017). Concurrent adversities and deliberate self-harm among indigenous Sami and majority Norwegian adolescents: the Norwegian Arctic Adolescent Health Study. SCANDINAVIAN JOURNAL OF CHILD AND ADOLESCENT PSYCHIATRY AND PSYCHOLOGY, 5(3), 92-103

B. Reigstad and S. Kvernmo. (2017). Concurrent adversities and suicide attempts among Sami and non-Sami adolescents: the Norwegian Arctic Adolescent Study (NAAHS). Nordic Journal of Psychiatry, 71(6), 425-432

B. S. M. Haugland. (2003). Paternal Alcohol Abuse: Relationship between Child Adjustment, Parental Characteristics, and Family Functioning. Child Psychiatry and Human Development, 34(2), 127-146

B. Sivertsen, A. G. Harvey, M. Gradisar, S. Pallesen and M. Hysing. (2021). Delayed sleep-wake phase disorder in young adults: prevalence and correlates from a national survey of Norwegian university students. Sleep Medicine, 77, 184-191

C. Aslund, K. W. Nilsson, B. Starrin and R. L. Sjoberg. (2007). Shaming experiences and the association between adolescent depression and psychosocial risk factors. European Child & Adolescent Psychiatry, 16(5), 298-304

C. Bjorkenstam, K. Kosidou and E. Bjorkenstam. (2017). Childhood adversity and risk of suicide: cohort study of 548721 adolescents and young adults in Sweden. BMJ (Clinical research ed.), 357, j1334

C. Bortes, M. Strandh and K. Nilsson. (2020). Parental Illness and Young People’s Education. Child Indicators Research, 13(6), 2069-2091

C. D. Hansen, M. J. Kirkeby, K. G. Kjelmann, J. H. Andersen and R. J. Moberg. (2021). The importance of adverse childhood experiences for labour market trajectories over the life course: a longitudinal study. BMC public health, 21(1), 2044

C. Holst, J. S. Tolstrup and U. Becker. (2022). Risk of somatic disease and mortality in individuals of parents with alcohol use disorder: a register-based cohort study. ADDICTION, 117(4), 905-912

C. Holst, J. S. Tolstrup, H. J. Sorensen and U. Becker. (2020). Family structure and alcohol use disorder: a register-based cohort study among offspring with and without parental alcohol use disorder. Addiction (Abingdon, England), 115(8), 1440-1449

C. Holst, J. S. Tolstrup, H. J. Sorensen, V. S. C. Pisinger and U. Becker. (2019). Parental alcohol use disorder with and without other mental disorders and offspring alcohol use disorder. Acta psychiatrica Scandinavica, 139(6), 508-517

C. Liu, A. Grotta, A. Hiyoshi, L. Berg and M. Rostila. (2022). School Outcomes Among Children Following Death of a Parent. JAMA network open, 5(4), e223842

C. Mohr-Jensen, C. M. Bisgaard, S. K. Boldsen and H.-C. Steinhausen. (2019). Attention-deficit/hyperactivity disorder in childhood and adolescence and the risk of crime in young adulthood in a Danish nationwide study. Journal of the American Academy of Child & Adolescent Psychiatry, 58(4), 443-452

C. S. Rasmussen, L. G. Nielsen, D. J. Petersen, E. Christiansen and N. Bilenberg. (2014). Adverse life events as risk factors for behavioural and emotional problems in a 7-year follow-up of a population-based child cohort. Nordic Journal of Psychiatry, 68(3), 189-195

C. W. Appel, C. Johansen, J. Christensen, K. Frederiksen, H. Hjalgrim, S. O. Dalton, A. Dencker, J. Dige, P. Boge, A. Dyregrov, O. A. Mikkelsen, L. W. Lund, M. T. Hoybye and P. E. Bidstrup. (2016). Risk of Use of Antidepressants Among Children and Young Adults Exposed to the Death of a Parent. Epidemiology (Cambridge, Mass.), 27(4), 578-85

C. W. Appel, K. Frederiksen, H. Hjalgrim, A. Dyregrov, S. O. Dalton, A. Dencker, M. T. Hoybye, J. Dige, P. Boge, O. A. Mikkelsen, C. Johansen and P. Envold Bidstrup. (2019). Depressive symptoms and mental health-related quality of life in adolescence and young adulthood after early parental death. Scandinavian journal of public health, 47(7), 782-792

C. Wildeman and S. H. Andersen. (2017). PATERNAL INCARCERATION AND CHILDREN'S RISK OF BEING CHARGED BY EARLY ADULTHOOD: EVIDENCE FROM A DANISH POLICY SHOCK. CRIMINOLOGY, 55(1), 32-58

C. Wildeman, S. H. Andersen, H. Lee and K. B. Karlson. (2014). Parental incarceration and child mortality in Denmark. American journal of public health, 104(3), 428-433

D. A. Winther-Lindqvist. (2014). UNCERTAINTY AS ORGANIZING PRINCIPLE OF ACTION--TEENAGERS LIVING WITH PARENTAL SOMATIC ILLNESS. Illness, Crisis & Loss, 22(2), 95-113

D. Paksarian, W. W. Eaton, P. B. Mortensen, K. R. Merikangas and C. B. Pedersen. (2015). A population-based study of the risk of schizophrenia and bipolar disorder associated with parent-child separation during development. Psychological Medicine, 45(13), 2825-2837

E. Agerbo, M. Nordentoft and P. B. Mortensen. (2002). Familial, psychiatric, and socioeconomic risk factors for suicide in young people: nested case-control study. BMJ-BRITISH MEDICAL JOURNAL, 325(7355), 74-77

E. B. Ahmad-Nielsen, N. G. Andersen, K. Andersen, A. S. Nielsen and A. I. Mellentin. (2019). The degree of multidimensional severity of alcohol use disorder among treatment-seeking patients: Is there an additive effect of parental alcohol use disorder? European Addiction Research, 25(4), 191-197

E. Bjorkenstam, A. Hjern and B. Vinnerljung. (2017). Adverse childhood experiences and disability pension in early midlife: results from a Swedish National Cohort Study. European journal of public health, 27(3), 472-477

E. Bjorkenstam, A. Hjern, C. Bjorkenstam and K. Kosidou. (2018). Association of Cumulative Childhood Adversity and Adolescent Violent Offending With Suicide in Early Adulthood. JAMA psychiatry, 75(2), 185-193

E. Bjorkenstam, A. Hjern, E. Mittendorfer-Rutz, B. Vinnerljung, J. Hallqvist and R. Ljung. (2013). Multi-Exposure and Clustering of Adverse Childhood Experiences, Socioeconomic Differences and Psychotropic Medication in Young Adults. PLoS ONE, 8(1), e53551

E. Bjorkenstam, B. Burstrom, A. Hjern, B. Vinnerljung, K. Kosidou and L. Berg. (2019). Cumulative childhood adversity, adolescent psychiatric disorder and violent offending in young adulthood. European Journal of Public Health, 29(5), 855-861

E. Bjorkenstam, B. Burstrom, B. Vinnerljung and K. Kosidou. (2016). Childhood adversity and psychiatric disorder in young adulthood: An analysis of 107,704 Swedes. Journal of Psychiatric Research, 77, 67-75

E. Bjorkenstam, B. Vinnerljung and A. Hjern. (2017). Impact of childhood adversities on depression in early adulthood: A longitudinal cohort study of 478,141 individuals in Sweden. Journal of Affective Disorders, 223, 95-100

E. Bjorkenstam, C. Bjorkenstam, B. Jablonska and K. Kosidou. (2018). Cumulative exposure to childhood adversity, and treated attention deficit/hyperactivity disorder: a cohort study of 543 650 adolescents and young adults in Sweden. Psychological medicine, 48(3), 498-507

E. Bjorkenstam, C. Dalman, B. Vinnerljung, G. R. Weitoft, D. J. Walder and B. Burstrom. (2016). Childhood household dysfunction, school performance and psychiatric care utilisation in young adults: A register study of 96 399 individuals in Stockholm County. Journal of Epidemiology and Community Health, 70(5), 473-480

E. Bjorkenstam, K. Kosidou and C. Bjorkenstam. (2016). Childhood household dysfunction and risk of self-harm: A cohort study of 107 518 young adults in Stockholm County. International Journal of Epidemiology, 45(2), 501-511

E. Bjorkenstam, L. Ekselius, B. Burstrom, K. Kosidou and C. Bjorkenstam. (2017). Association between childhood adversity and a diagnosis of personality disorder in young adulthood: a cohort study of 107,287 individuals in Stockholm County. European Journal of Epidemiology, 32(8), 721-731

E. Bjorkenstam, M. Helgesson and E. Mittendorfer-Rutz. (2021). Childhood adversity and common mental disorders in young employees in Sweden: is the association affected by early adulthood occupational class? Social psychiatry and psychiatric epidemiology, 56(2), 237-246

E. C. Long, S. L. Lonn, J. Sundquist, K. Sundquist and K. S. Kendler. (2018). The role of parent and offspring sex on risk for externalizing psychopathology in offspring with parental alcohol use disorder: a national Swedish study. Social psychiatry and psychiatric epidemiology, 53(12), 1381-1389

E. Christiansen, K. J. Larsen, E. Agerbo, N. Bilenberg and E. Stenager. (2013). Incidence and risk factors for suicide attempts in a general population of young people: A Danish register-based study. Australian and New Zealand Journal of Psychiatry, 47(3), 259-270

E. Fransson, J. Turunen, A. Hjern, V. Ostberg and M. Bergstrom. (2016). Psychological complaints among children in joint physical custody and other family types: Considering parental factors. Scandinavian journal of public health, 44(2), 177-183

E. Fransson, S. B. Laftman, V. Ostberg, A. Hjern and M. Bergstrom. (2018). The Living Conditions of Children with Shared Residence - the Swedish Example. Child indicators research, 11(3), 861-883

E. Hammar, M. Bladh and S. Agnafors. (2020). Mental health and experience of being bullied in 12-year-old children with overweight and obesity. ACTA PAEDIATRICA, 109(7), 1450-1457

E. Just-Ostergaard, E. L. Mortensen and T. Flensborg-Madsen. (2018). Major life events and risk of alcohol use disorders: A prospective cohort study. Addiction, 113(1), 25-33

E. K. Kallander, B. M. Weimand, K. Hanssen‐Bauer, B. Van Roy and T. Ruud. (2021). Factors associated with quality of life for children affected by parental illness or substance abuse. Scandinavian Journal of Caring Sciences, 35(2), 405-419

E. K. Kallander, B. Weimand, T. Ruud, S. Becker, B. Van Roy and K. Hanssen-Bauer. (2018). Outcomes for children who care for a parent with a severe illness or substance abuse. Child & Youth Services, 39(4), 228-249

E. Keskinen, R. Marttila, H. Koivumaa-Honkanen, K. Moilanen, S. Keinanen-Kiukaanniemi, M. Timonen, M. Isohanni, J. McGrath, J. Miettunen and E. Jaaskelainen. (2018). Search for protective factors for psychosis - a population-based sample with special interest in unaffected individuals with parental psychosis. EARLY INTERVENTION IN PSYCHIATRY, 12(5), 869-878

E. Kjelsberg and C. Friestad. (2008). Social adversities in first-time and repeat prisoners. International Journal of Social Psychiatry, 54(6), 514-526

E. L. W. Nordh, G. Priebe, K. Grip, M. Afzelius and U. Axberg. (2022). Mental health in children of parents being treated by specialised psychiatric services. Scandinavian journal of public health, 14034948221076208

E. M. Wiig, B. S. M. Haugland, A. Halsa and S. M. Myhra. (2017). Substance-dependent women becoming mothers: breaking the cycle of adverse childhood experiences. Child & Family Social Work, 22(1), 26-35

E. Porthan, M. Lindberg, E. Ekholm, N. M. Scheinin, L. Karlsson, H. Karlsson and J. Harkonen. (2020). Parental divorce in childhood does not independently predict maternal depressive symptoms during pregnancy. BMC Pregnancy and Childbirth, 20(1), 520

E. Tedgard and M. Rastam. (2016). Vulnerable parenting among mothers with substance abuse in their family of origin: a cross-sectional comparative study of mothers in an infant and toddler program. SpringerPlus, 5(1), 1540

F. A. Torvik, K. Rognmo, H. Ask, E. Roysamb and K. Tambs. (2011). Parental alcohol use and adolescent school adjustment in the general population: results from the HUNT study. BMC public health, 11, 706

F. Gullbra, T. Smith-Sivertsen, A. H. Graungaard, G. Rortveit and M. Hafting. (2016). How can the general practitioner support adolescent children of ill or substance-abusing parents? A qualitative study among adolescents. Scandinavian Journal of Primary Health Care, 34(4), 360-367

F. H. Jonsson, U. Njardvik, G. Olafsdottir and S. J. Gretarsson. (2000). Parental divorce: Long-term effects on mental health, family relations and adult sexual behavior. SCANDINAVIAN JOURNAL OF PSYCHOLOGY, 41(2), 101-105

F. Lindblad, G. R. Weitoft and A. Hjern. (2011). Maternal and paternal psychopathology increases risk of offspring ADHD equally. Epidemiology and Psychiatric Sciences, 20(4), 367-372

F. Naevdal and F. Thuen. (2004). Residence arrangements and well-being: A study of Norwegian adolescents. SCANDINAVIAN JOURNAL OF PSYCHOLOGY, 45(5), 363-371

F. Steele, W. Sigle-Rushton and O. Kravdal. (2009). Consequences of family disruption on children's educational outcomes in Norway. Demography, 46(3), 553-574

F. Thuen, K. Breivik, B. Wold and G. Ulveseter. (2015). Growing Up with One or Both Parents: The Effects on Physical Health and Health-Related Behavior Through Adolescence and into Early Adulthood. Journal of Divorce & Remarriage, 56(6), 451-474

G. N. Giordano, H. Ohlsson, K. S. Kendler, K. Sundquist and J. Sundquist. (2014). Unexpected adverse childhood experiences and subsequent drug use disorder: a Swedish population study (1995-2011). Addiction, 109(7), 1119-1127

G. Olsson and J. Fritzell. (2017). Family Composition and Youth Health Risk Behaviors: the Role of Patental Relation and the School Context. CHILD INDICATORS RESEARCH, 10(2), 403-421

G. R. Parra, I. Patwardhan, W. A. Mason, M. B. Chmelka, J. Savolainen, J. Miettunen and M.-R. Jarvelin. (2020). Parental Alcohol Use and the Alcohol Misuse of their Offspring in a Finnish Birth Cohort: Investigation of Developmental Timing. Journal of youth and adolescence, 49(8), 1702-1715

G. R. Weitoft, A. Hjern and M. Rosen. (2004). School's out! Why earlier among children of lone parents? INTERNATIONAL JOURNAL OF SOCIAL WELFARE, 13(2), 134-144

G. R. Weitoft, A. Hjern, B. Haglund and M. Rosen. (2003). Mortality, severe morbidity, and injury in children living with single parents in Sweden: A population-based study. Lancet, 361(9354), 289-295

G. Ulveseter, K. Breivik and F. Thuen. (2010). Health-related adjustment of adolescents in various postdivorce family structures with main focus on father custody with and without a stepmother. Journal of Divorce & Remarriage, 51(7), 379-395

H. B. Danielsdottir, T. Aspelund, E. B. Thordardottir, K. Fall, F. Fang, G. Tomasson, H. Runarsdottir, Q. Yang, K. W. Choi, B. Kennedy, T. Halldorsdottir, D. Lu, H. Song, J. Jakobsdottir, A. Hauksdottir and U. A. Valdimarsdottir. (2022). Adverse childhood experiences and resilience among adult women: A population-based study. eLife, 11, e71770

H. Bould, I. Koupil, C. Dalman, B. DeStavola, G. Lewis and C. Magnusson. (2015). Parental mental illness and eating disorders in offspring. International Journal of Eating Disorders, 48(4), 383-391

H. C. Wilcox, S. J. Kuramoto, P. Lichtenstein, N. Langstrom, D. A. Brent and B. Runeson. (2010). Psychiatric Morbidity, Violent Crime, and Suicide Among Children and Adolescents Exposed to Parental Death. JOURNAL OF THE AMERICAN ACADEMY OF CHILD AND ADOLESCENT PSYCHIATRY, 49(5), 514-523

H. Elonheimo, A. Sourander, S. Niemela and H. Helenius. (2011). Generic and crime type specific correlates of youth crime: a Finnish population-based study. Social psychiatry and psychiatric epidemiology, 46(9), 903-14

H. Elonheimo, A. Sourander, S. Niemela, A.-M. Nuutila, H. Helenius, L. Sillanmaki, T. Ristkari and K. Parkkola. (2009). Psychosocial correlates of police-registered youth crime. A Finnish population-based study. Nordic journal of psychiatry, 63(4), 292-300

H. J. Sorensen, A. M. Manzardo, J. Knop, E. C. Penick, W. Madarasz, E. J. Nickel, U. Becker and E. L. Mortensen. (2011). The Contribution of Parental Alcohol Use Disorders and Other Psychiatric Illness to the Risk of Alcohol Use Disorders in the Offspring. Alcoholism: Clinical and Experimental Research, 35(7), 1315-1320

H. Kristiansen, M. Roelants, R. Bjerknes and P. B. Juliusson. (2020). Norwegian children and adolescents in blended families are at risk of larger one-year BMI increments. Acta Paediatrica, International Journal of Paediatrics, 109(3), 587-594

H. Krona, M. Nyman, H. Andreasson, N. Vicencio, H. Anckarsater, M. Wallinius, T. Nilsson and B. Hofvander. (2017). Mentally disordered offenders in Sweden: differentiating recidivists from non-recidivists in a 10-year follow-up study. Nordic Journal of Psychiatry, 71(2), 102-109

H. L. F. Eriksen, C. Hvidtfeldt and H. B. Lilleor. (2017). Family Disruption and Social, Emotional and Behavioral Functioning in Middle Childhood. JOURNAL OF CHILD AND FAMILY STUDIES, 26(4), 1077-1089

H. Oldrup. (2019). 'So we try to do normal things, like a family': sociologies of children in the prison visiting room. FAMILIES RELATIONSHIPS AND SOCIETIES, 8(2), 303-319

H. Pihkala, A. Cederstrom and M. Sandlund. (2011). The family members' experiences of the family talk intervention. European Child and Adolescent Psychiatry, 20, S35

H. Pihkala, M. Sandlund and A. Cederstrom. (2012). Children in Beardslee's family intervention: Relieved by understanding of parental mental illness. International Journal of Social Psychiatry, 58(6), 623-628

H. Pihkala, N. Dimova-Branstrom and M. Sandlund. (2017). Talking about parental substance abuse with children: eight families' experiences of Beardslee's family intervention. Nordic journal of psychiatry, 71(5), 395-401

H. Remes, H. Moustgaard, L. M. Kestila and P. Martikainen. (2019). Parental education and adolescent health problems due to violence, self-harm and substance use: what is the role of parental health problems? JOURNAL OF EPIDEMIOLOGY AND COMMUNITY HEALTH, 73(3), 225-231

H. Varis, M. Hagnas, I. Mikkola, T. Nordstrom, K. Puukka, A. Taanila and S. Keinanen-Kiukaanniemi. (2021). Parental separation and offspring morbidity in adulthood: a descriptive study of the Northern Finland Birth Cohort 1966. Scandinavian journal of public health, 14034948211014296

H. Vuorimaa, K. Tamm, V. Honkanen, E. Komulainen, Y. T. Konttinen and N. Santavirta. (2011). Pain in juvenile idiopathic arthritis-A family matter. Children's Health Care, 40(1), 34-52

H. Zeratsion, C. B. Bjertness, E. Bjertness, M. Dalsklev, O. R. Haavet, J. A. Halvorsen, L. Lien and B. Claussen. (2015). The influence of parental divorce on educational ambitions of 18/19 year-old adolescents from Oslo, Norway. Journal of Child and Family Studies, 24(10), 2865-2873

H. Zeratsion, C. B. Bjertness, L. Lien, O. R. Haavet, M. Dalsklev, J. A. Halvorsen, E. Bjertness and B. Claussen. (2014). Does parental divorce increase risk behaviors among 15/16 and 18/19 year-old adolescents? A study from Oslo, Norway. Clinical Practice and Epidemiology in Mental Health, 10(1), 59-66

H. Zeratsion, M. Dalsklev, E. Bjertness, L. Lien, O. R. Haavet, J. A. Halvorsen, C. B. Bjertness and B. Claussen. (2013). Parental divorce in late adolescence does not seem to increase mental health problems: a population study from Norway. BMC public health, 13, 413

H.-M. Husu and M. Ylilahti. (2020). The formation of long-term unemployed young adults' emotional capital during childhood: A Bourdieusian approach to emotional harm. Journal of Youth Studies, 23(9), 1161-1177

I. D. Sigfusdottir, B. B. Asgeirsdottir, J. F. Sigurdsson and G. H. Gudjonsson. (2011). Physical activity buffers the effects of family conflict on depressed mood: A study on adolescent girls and boys. JOURNAL OF ADOLESCENCE, 34(5), 895-902

I. Haahr-Pedersen, P. Hyland, M. Hansen, C. Perera, P. Spitz, R. H. Bramsen and F. Vallieres. (2021). Patterns of childhood adversity and their associations with internalizing and externalizing problems among at-risk boys and girls. Child Abuse and Neglect, 121, 105272

I. O. Lund, S. Skurtveit, M. Handal, A. Bukten, F. A. Torvik, E. Ystrom and J. B. Andreas. (2019). Association of Constellations of Parental Risk With Children's Subsequent Anxiety and Depression Findings From a HUNT Survey and Health Registry Study. JAMA PEDIATRICS, 173(3), 251-259

I. Prix and J. Erola. (2017). Does death really make us equal? Educational attainment and resource compensation after paternal death in Finland. SOCIAL SCIENCE RESEARCH, 64, 171-183

I. Ranoyen, C. A. Klockner, J. Wallander and T. Jozefiak. (2015). Associations between internalizing problems in adolescent daughters versus sons and mental health problems in mothers versus fathers (the HUNT study). Journal of Child and Family Studies, 24(7), 2008-2020

I. Rossow and G. Lauritzen. (2001). Shattered childhood: A key issue in suicidal behavior among drug addicts? Addiction, 96(2), 227-240

I. S. Jakobsen and E. Christiansen. (2011). Young people's risk of suicide attempts in relation to parental death: A population-based register study. Journal of Child Psychology and Psychiatry, 52(2), 176-183

I. Storksen, A. A. Thorsen, K. Overland and S. R. Brown. (2012). Experiences of Daycare Children of Divorce. Early Child Development and Care, 182(7), 807-825

I. Storksen, E. Roysamb, H. K. Gjessing, T. Moum and K. Tambs. (2007). Marriages and psychological distress among adult offspring of divorce: A Norwegian study. Scandinavian Journal of Psychology, 48(6), 467-476

I. Storksen, E. Roysamb, T. L. Holmen and K. Tambs. (2006). Adolescent adjustment and well-being: effects of parental divorce and distress. Scandinavian journal of psychology, 47(1), 75-84

I. Storksen, E. Roysamb, T. Moum and K. Tambs. (2005). Adolescents with a childhood experience of parental divorce: A longitudinal study of mental health and adjustment. Journal of Adolescence, 28(6), 725-739

I. Weiber, P.-A. Tengland, J. S. Berglund and M. Eklund. (2020). Everyday life when growing up with a mother with an intellectual or developmental disability: Four retrospective life-stories. Scandinavian Journal of Occupational Therapy, 27(6), 418-430

J. A. Ronning, A. Haavisto, G. Nikolakaros, H. Helenius, T. Tamminen, I. Moilanen, K. Kumpulainen, J. Piha, F. Almqvist and A. Sourander. (2011). Factors associated with reported childhood depressive symptoms at age 8 and later self-reported depressive symptoms among boys at age 18. Social psychiatry and psychiatric epidemiology, 46(3), 207-18

J. Burdzovic Andreas, F. Ask Torvik, E. Ystrom, S. Skurtveit, M. Handal, P. Martinez, A.-M. Laslett and I. O. Lund. (2021). Parental risk constellations and future alcohol use disorder (AUD) in offspring: A combined HUNT survey and health registries study. Psychology of Addictive Behaviors, No-Specified

J. Drevin, J. Stern, E. M. Annerback, M. Peterson, S. Butler, T. Tyden, A. Berglund, M. Larsson and P. Kristiansson. (2015). Adverse childhood experiences influence development of pain during pregnancy. Acta Obstetricia et Gynecologica Scandinavica, 94(8), 840-846

J. E. Salvatore, S. Larsson Lonn, E. C. Long, J. Sundquist, K. S. Kendler, K. Sundquist and A. C. Edwards. (2019). Parental alcohol use disorder and offspring marital outcomes. Addiction, 114(1), 81-91

J. Erman and J. Harkonen. (2017). Parental Separation and School Performance Among Children of Immigrant Mothers in Sweden. European journal of population = Revue europeenne de demographie, 33(2), 267-292

J. Isaksson, K. Nilsson and F. Lindblad. (2013). Early psychosocial adversity and cortisol levels in children with attention-deficit/hyperactivity disorder. European Child & Adolescent Psychiatry, 22(7), 425-432

J. Korkeila, J. Vahtera, K. Korkeila, M. Kivimaki, M. Sumanen, K. Koskenvuo and M. Koskenvuo. (2010). Childhood adversities as predictors of incident coronary heart disease and cerebrovascular disease. Heart, 96(4), 298-303

J. Korkeila, R. Lietzen, L. H. Sillanmaki, P. Rautava, K. Korkeila, M. Kivimaki, M. Koskenvuo and J. Vahtera. (2012). Childhood adversities and adult-onset asthma: A cohort study. BMJ Open, 2(5), e001625

J. Kaasboll, N. Skokauskas, S. Lydersen and A. M. Sund. (2021). Parental Chronic Illness, Internalizing Problems in Young Adulthood and the Mediating Role of Adolescent Attachment to Parents: A Prospective Cohort Study. Frontiers in Psychiatry, 12, 807563

J. Landberg, A. K. Danielsson and T. Hemmingsson. (2019). Fathers' alcohol use and suicidal behaviour in offspring during youth and young adulthood. Acta Psychiatrica Scandinavica, 140(6), 563-573

J. Li, J. Olsen, M. Vestergaard, C. Obel, J. L. Baker and T. I. A. Sorensen. (2012). Bereavement in early life and later childhood overweight. Obesity facts, 5(6), 881-9

J. Li, M. Vestergaard, S. Cnattingius, M. Gissler, B. H. Bech, C. Obel and J. Olsen. (2014). Mortality after Parental Death in Childhood: A Nationwide Cohort Study from Three Nordic Countries. PLOS MEDICINE, 11(7)

J. Marcussen, L. Hounsgaard, M. O'Connor, S. Moller, R. Wilson and F. Thuen. (2021). Parental death in young adults with divorced compared to non-divorced parents: The effect on prolonged grief and mental health. Death studies, 45(6), 437-450

J. Pitkanen, H. Remes, H. Moustgaard and P. Martikainen. (2021). Parental socioeconomic resources and adverse childhood experiences as predictors of not in education, employment, or training: A Finnish register-based longitudinal study. Journal of Youth Studies, 24(1), 1-18

J. Pitkanen, H. Remes, M. Aaltonen and P. Martikainen. (2019). Experience of maternal and paternal adversities in childhood as determinants of self-harm in adolescence and young adulthood. Journal of epidemiology and community health, 73(11), 1040-1046

J. Salmela, E. Mauramo, T. Lallukka, O. Rahkonen and N. Kanerva. (2019). Associations between Childhood Disadvantage and Adult Body Mass Index Trajectories: A Follow-Up Study among Midlife Finnish Municipal Employees. Obesity Facts, 12(5), 564-574

J. Sveen, U. Kreicbergs, U. Melcher and A. Alvariza. (2016). Teenagers' reasoning about a parent's recent death in cancer. Palliative & supportive care, 14(4), 349-357

J. T. Larsen, T. Munk-Olsen, C. M. Bulik, L. M. Thornton, S. V. Koch, P. B. Mortensen and L. Petersen. (2017). Early childhood adversities and risk of eating disorders in women: A Danish register-based cohort study. International Journal of Eating Disorders, 50(12), 1404-1412

J. Turunen, E. Fransson and M. Bergstrom. (2017). Self-esteem in children in joint physical custody and other living arrangements. Public Health, 149, 106-112

J. Turunen. (2014). Adolescent Educational Outcomes in Blended Families: Evidence from Swedish Register Data. Journal of Divorce & Remarriage, 55(7), 568-589

J. Turunen. (2017). Shared Physical Custody and Children’s Experience of Stress. Journal of Divorce & Remarriage, 58(5), 371-392

J. Y. Moberg, D. Larsen and A. Brødsgaard. (2017). Striving for balance between caring and restraint: young adults' experiences with parental multiple sclerosis. Journal of Clinical Nursing (John Wiley & Sons, Inc.), 26(9), 1363-1374

J. Y. Moberg, M. Magyari, N. Koch-Henriksen, L. C. Thygesen, B. Laursen and P. Soelberg Sorensen. (2016). Educational achievements of children of parents with multiple sclerosis: A nationwide register-based cohort study. Journal of Neurology, 263(11), 2229-2237

J.-K. Vederhus, S. H. Haugland and C. Timko. (2022). A mediational analysis of adverse experiences in childhood and quality of life in adulthood. International journal of methods in psychiatric research, 31(1), e1904

K. A. Davidsen, E. Christiansen, D. Haubek, J. Asmussen, A. Ranning, A. A. E. Thorup, M. Nordentoft, S. Harder and N. Bilenberg. (2021). Parental mental illness, attendance at preventive child healthcare and dental caries in the offspring: a nation-wide population-based cohort study. Social psychiatry and psychiatric epidemiology, 56(4), 583-592

K. Appelqvist-Schmidlechner, M. Henriksson, M. Joukamaa, K. Parkkola, M. Upanne and E. Stengard. (2011). Psychosocial factors associated with suicidal ideation among young men exempted from compulsory military or civil service. Scandinavian journal of public health, 39(8), 870-9

K. Appelqvist-Schmidlechner, M. Upanne, M. Henriksson, K. Parkkola and E. Stengard. (2010). Young men exempted from compulsory military or civil service in Finland-A group of men in need of psychosocial support? Scandinavian Journal of Public Health, 38(2), 168-176

K. Barclay and M. Hallsten. (2022). Does the impact of parental death vary by parental socioeconomic status? A study of children's educational and occupational attainment. Journal of Marriage and Family, 84(1), 141-164

K. Breivik and D. Olweus. (2006). Children of divorce in a Scandinavian welfare state: are they less affected than US children? Scandinavian journal of psychology, 47(1), 61-74

K. Breivik, D. Olweus and I. Endresen. (2009). Does the quality of parent--child relationships mediate the increased risk for antisocial behavior and substance use among adolescents in single-mother and single-father families? Journal of Divorce & Remarriage, 50(6), 400-426

K. Gauffin, A. Hjern, B. Vinnerljung and E. Bjorkenstam. (2016). Childhood Household Dysfunction, Social Inequality and Alcohol Related Illness in Young Adulthood. A Swedish National Cohort Study. PloS one, 11(3), e0151755

K. Harkonmäki, K. Korkeila, J. Vahtera, M. Kivimäki, S. Suominen, L. Sillanmäki and M. Koskenvuo. (2007). Childhood adversities as a predictor of disability retirement. Journal of Epidemiology & Community Health, 61(6), 479-484

K. J. Tsuchiya, E. Agerbo and P. B. Mortensen. (2005). Parental death and bipolar disorder: A robust association was found in early maternal suicide. Journal of Affective Disorders, 86(2), 151-159

K. Korkeila, S.-L. Kivela, S. Suominen, J. Vahtera, M. Kivimaki, J. Sundell, H. Helenius and M. Koskenvuo. (2004). Childhood adversities, parent-child relationships and dispositional optimism in adulthood. Social Psychiatry and Psychiatric Epidemiology: The International Journal for Research in Social and Genetic Epidemiology and Mental Health Services, 39(4), 286-292

K. Koskenvuo and M. Koskenvuo. (2015). Childhood adversities predict strongly the use of psychotropic drugs in adulthood: a population-based cohort study of 24,284 Finns. Journal of epidemiology and community health, 69(4), 354-360

K. M. Haatainen, A. Tanskanen, J. Kylma, K. Honkalampi, H. Koivumaa-Honkanen, J. Hintikka, R. Antikainen and H. Viinamaki. (2003). Gender differences in the association of adult hopelessness with adverse childhood experiences. Social Psychiatry and Psychiatric Epidemiology: The International Journal for Research in Social and Genetic Epidemiology and Mental Health Services, 38(1), 12-17

K. Moxnes. (2003). Risk factors in divorce: perceptions by the children involved. Childhood, 10(2), 131-146

K. Pape, W. Cowell, C. S. Sejbaek, N. W. Andersson, C. Svanes, H. A. Kolstad, X. Liu, K. S. Hougaard, R. J. Wright and V. Schlunssen. (2021). Adverse childhood experiences and asthma: Trajectories in a national cohort. Thorax, 76(6), 547-553

K. Raitasalo and M. Holmila. (2017). Parental substance abuse and risks to children's safety, health and psychological development. Drugs: Education, Prevention & Policy, 24(1), 17-22

K. Raitasalo, J. Ostergaard and S. B. Andrade. (2021). Educational attainment by children with parental alcohol problems in Denmark and Finland. Nordic Studies on Alcohol and Drugs, 38(3), 227-242

K. Raitasalo, M. Holmila, I. Autti-Ramo, I. L. Notkola and H. Tapanainen. (2015). Hospitalisations and out-of-home placements of children of substance-abusing mothers: A register-based cohort study. DRUG AND ALCOHOL REVIEW, 34(1), 38-45

K. Raitasalo, M. Holmila, M. Jaaskelainen and P. Santalahti. (2019). The effect of the severity of parental alcohol abuse on mental and behavioural disorders in children. European Child and Adolescent Psychiatry, 28(7), 913-922

K. Rognmo, F. A. Torvik, H. Ask, E. Roysamb and K. Tambs. (2012). Paternal and maternal alcohol abuse and offspring mental distress in the general population: the Nord-Trondelag health study. BMC public health, 12, 448

K. S. Havnen, K. Breivik, K. M. Stormark and R. Jakobsen. (2011). Why do children placed out-of-home because of parental substance abuse have less mental health problems than children placed for other reasons? Children and Youth Services Review, 33(10), 2010-2017

K. S. Kendler, H. Ohlsson, J. Sundquist and K. Sundquist. (2020). The Rearing Environment and Risk for Major Depression: A Swedish National High-Risk Home-Reared and Adopted-Away Co-Sibling Control Study. American Journal of Psychiatry, 177(5), 447-453

K. S. Kendler, H. Ohlsson, K. Sundquist and J. Sundquist. (2014). Peer deviance, parental divorce, and genetic risk in the prediction of drug abuse in a nationwide Swedish sample: Evidence of environment-environment and gene-environment interaction. JAMA Psychiatry, 71(4), 439-445

K. S. Kendler, H. Ohlsson, K. Sundquist and J. Sundquist. (2016). The rearing environment and risk for drug abuse: a Swedish national high-risk adopted and not adopted co-sibling control study. Psychological medicine, 46(7), 1359-1366

K. S. Kendler, H. Ohlsson, S. Bacanu, J. Sundquist and K. Sundquist. (2020). The risk for drug abuse, alcohol use disorder, and psychosocial dysfunction in offspring from high-density pedigrees: its moderation by personal, family, and community factors. Molecular psychiatry, 25(8), 1777-1786

K. S. Kendler, K. Sundquist, H. Ohlsson, K. Palmer, H. Maes, M. A. Winkleby and J. Sundquist. (2012). Genetic and familial environmental influences on the risk for drug abuse: A national Swedish adoption study. Archives of General Psychiatry, 69(7), 690-697

L. B. Lydsdottir, L. M. Howard, H. Olafsdottir, H. Einarsson, T. Steingrimsdottir and J. F. Sigurdsson. (2019). Adverse life experiences and common mental health problems in pregnancy: a causal pathway analysis. Archives of Women's Mental Health, 22(1), 75-83

L. Berg, M. Rostila and A. Hjern. (2016). Parental death during childhood and depression in young adults-A national cohort study. Journal of Child Psychology and Psychiatry, 57(9), 1092-1098

L. Berg, M. Rostila, A. Arat and A. Hjern. (2019). Parental death during childhood and violent crime in late adolescence to early adulthood: a Swedish national cohort study. PALGRAVE COMMUNICATIONS, 5

L. Berg, M. Rostila, J. Saarela and A. Hjern. (2014). Parental death during childhood and subsequent school performance. Pediatrics, 133(4), 682-689

L. Clemmensen, J. van Os, M. Drukker, A. Munkholm, M. K. Rimvall, M. Vaever, C. U. Rask, A. A. Bartels-Velthuis, A. M. Skovgaard and P. Jeppesen. (2016). Psychotic experiences and hyper-theory-of-mind in preadolescence-A birth cohort study. Psychological Medicine, 46(1), 87-101

L. Grotvedt, H. Stigum, R. Hovengen and S. Graff-Iversen. (2008). Social differences in smoking and snuff use among Norwegian adolescents: A population based survey. BMC Public Health, 8, 322

L. H. Andersen. (2016). How Children's Educational Outcomes and Criminality Vary by Duration and Frequency of Paternal Incarceration. ANNALS OF THE AMERICAN ACADEMY OF POLITICAL AND SOCIAL SCIENCE, 665(1), 149-170

L. Hanberger, J. Ludvigsson and S. Nordfeldt. (2009). Health-related quality of life in intensively treated young patients with type 1 diabetes. Pediatric Diabetes, 10(6), 374-381

L. Hohwü, M. Gissler, A. Sjöberg, A. M. Biehl, A. L. Kristjansson and C. Obel. (2014). Prevalence of overweight in 2 to 17 year-old children and adolescents whose parents live separately: A Nordic cross-sectional study. BMC Public Health, 14

L. Kaskeala, L. Sillanmaki and A. Sourander. (2015). Help-seeking behaviour among Finnish adolescent males. Nordic Journal of Psychiatry, 69(8), 605-612

L. Kestila, O. Rahkonen, T. Martelin, M. Lahti-Koski and S. Koskinen. (2009). Do childhood social circumstances affect overweight and obesity in early adulthood? Scandinavian Journal of Public Health, 37(2), 206-219

L. Kestila, S. Koskinen, T. Martelin, O. Rahkonen, T. Pensola, H. Aro and A. Aromaa. (2006). Determinants of health in early adulthood: What is the role of parental education, childhood adversities and own education? European Journal of Public Health, 16(3), 305-314

L. Kestila, S. Koskinen, T. Martelin, O. Rahkonen, T. Pensola, S. Pirkola, K. Patja and A. Aromaa. (2006). Influence of parental education, childhood adversities, and current living conditions on daily smoking in early adulthood. European Journal of Public Health, 16(6), 617-626

L. Kestila, T. Martelin, O. Rahkonen, K. Joutsenniemi, S. Pirkola, K. Poikolainen and S. Koskinen. (2008). Childhood and current determinants of heavy drinking in early adulthood. Alcohol and Alcoholism, 43(4), 460-469

L. Kestila, T. Martelin, O. Rahkonen, T. Harkanen and S. Koskinen. (2009). The contribution of childhood circumstances, current circumstances and health behaviour to educational health differences in early adulthood. BMC Public Health, 9, 164

L. Khemiri, H. Larsson, R. Kuja-Halkola, B. M. D'Onofrio, P. Lichtenstein, N. Jayaram-Lindstrom and A. Latvala. (2020). Association of parental substance use disorder with offspring cognition: a population family-based study. Addiction (Abingdon, England), 115(2), 326-336

L. Kinnunen, M. Niemela, H. Hakko, J. Miettunen, M. Merikukka, V. Karttunen, T. Ristikari, M. Gissler and S. Rasanen. (2018). Psychiatric diagnoses of children affected by their parents' traumatic brain injury: the 1987 Finnish Birth Cohort study. BRAIN INJURY, 32(7), 933-940

L. Kinnunen, T. Nordstrom, M. Niemela, S. Rasanen, S. Whittle and J. Miettunen. (2021). Parental physical illnesses and their association with subsequent externalizing and internalizing symptoms in children. Journal of Child and Family Studies, No-Specified

L. Kinnunen, T. Nordstrom, M. Niemela, S. Rasanen, S. Whittle, M. Sawyer and J. Miettunen. (2020). Parental somatic illnesses and their association with prodromal symptoms of psychosis among offspring. Schizophrenia Research, 224, 190-192

L. Knutsson-Medin, B. Edlund and M. Ramklint. (2007). Experiences in a group of grown-up children of mentally ill parents. Journal of Psychiatric and Mental Health Nursing, 14(8), 744-752

L. L. Laursen, K. B. Madsen, C. Obel and L. Hohwu. (2019). Family dissolution and children's social well-being at school: A historic cohort study. BMC Pediatrics, 19(1), 449

L. Mather, V. Blom and P. Svedberg. (2014). Stressful and Traumatic Life Events are Associated with Burnout-A Cross-Sectional Twin Study. INTERNATIONAL JOURNAL OF BEHAVIORAL MEDICINE, 21(6), 899-907

L. Uusitalo-Malmivaara and J. E. Lehto. (2013). Social Factors Explaining Children's Subjective Happiness and Depressive Symptoms. SOCIAL INDICATORS RESEARCH, 111(2), 603-615

L. V. Burrell, L. Mehlum and P. Qin. (2020). Educational attainment in offspring bereaved by sudden parental death from external causes: A national cohort study from birth and throughout adulthood. Social Psychiatry and Psychiatric Epidemiology: The International Journal for Research in Social and Genetic Epidemiology and Mental Health Services, No-Specified

L. V. Burrell, L. Mehlum and P. Qin. (2021). Co-occurrence of psychosocial sequelae in bereaved offspring. Journal of Affective Disorders, 283, 325-328

L. V. Burrell, L. Mehlum and P. Qin. (2021). Parental death by external causes and risk of hospital-treated deliberate self-harm in bereaved offspring. European child & adolescent psychiatry, 30(4), 539-548

L. V. Burrell, L. Mehlum and P. Qin. (2021). Parental death by external causes during childhood and risk of psychiatric disorders in bereaved offspring. Child and adolescent mental health

L. Wichstrom, J. Belsky and T. S. Berg-Nielsen. (2013). Preschool predictors of childhood anxiety disorders: a prospective community study. Journal of child psychology and psychiatry, and allied disciplines, 54(12), 1327-36

L. Wichstrom, T. S. Berg-Nielsen, A. Angold, H. L. Egger, E. Solheim and T. H. Sveen. (2012). Prevalence of Psychiatric Disorders in Preschoolers. Journal of Child Psychology and Psychiatry, 53(6), 695-705

M. A. Sheikh, B. Abelsen and J. A. Olsen. (2016). Clarifying associations between childhood adversity, social support, behavioral factors, and mental health, health, and well-being in adulthood: A population-based study. Frontiers in Psychology, 7

M. A. Sheikh. (2018). Childhood adversities and chronic conditions: examination of mediators, recall bias and age at diagnosis. International journal of public health, 63(2), 181-192

M. A. Sheikh. (2018). Coloring of the past via respondent's current psychological state, mediation, and the association between childhood disadvantage and morbidity in adulthood. Journal of Psychiatric Research, 103, 173-181

M. A. Sheikh. (2018). Retrospectively reported childhood adversity is associated with asthma and chronic bronchitis, independent of mental health. Journal of Psychosomatic Research, 114, 50-57

M. Arendt, L. Sher, L. Fjordback, J. Brandholdt and P. Munk-Jorgensen. (2007). Parental alcoholism predicts suicidal behavior in adolescents and young adults with cannabis dependence. International Journal of Adolescent Medicine and Health, 19(1), 67-77

M. B. Guldin, J. Li, H. S. Pedersen, C. Obel, E. Agerbo, M. Gissler, S. Cnattingius, J. Olsen and M. Vestergaard. (2015). Incidence of Suicide Among Persons Who Had a Parent Who Died During Their Childhood A Population-Based Cohort Study. JAMA PSYCHIATRY, 72(12), 1227-1234

M. Bask, T. Ristikari, A. Hautakoski and M. Gissler. (2017). Psychiatric diagnoses as grounds for disability pension among former child welfare clients. LONGITUDINAL AND LIFE COURSE STUDIES, 8(4), 365-381

M. Bergstrom, B. Modin, E. Fransson, L. Rajmil, M. Berlin, P. A. Gustafsson and A. Hjern. (2013). Living in two homes-a Swedish national survey of wellbeing in 12 and 15 year olds with joint physical custody. BMC public health, 13, 868

M. Bergstrom, E. Fransson, A. Hjern, L. Kohler and T. Wallby. (2014). Mental health in Swedish children living in joint physical custody and their parents' life satisfaction: a cross-sectional study. Scandinavian journal of psychology, 55(5), 433-439

M. Bergstrom, E. Fransson, B. Modin, M. Berlin, P. A. Gustafsson and A. Hjern. (2015). Fifty moves a year: is there an association between joint physical custody and psychosomatic problems in children? Journal of epidemiology and community health, 69(8), 769-774

M. Bergstrom, E. Fransson, H. Fabian, A. Hjern, A. Sarkadi and R. Salari. (2018). Preschool children living in joint physical custody arrangements show less psychological symptoms than those living mostly or only with one parent. Acta Paediatrica, International Journal of Paediatrics, 107(2), 294-300

M. Bergstrom, E. Fransson, M. B. Wells, L. Kohler and A. Hjern. (2019). Children with two homes: Psychological problems in relation to living arrangements in Nordic 2- to 9-year-olds. Scandinavian journal of public health, 47(2), 137-145

M. Bergstrom, R. Salari, A. Hjern, R. Hognas, K. Bergqvist and E. Fransson. (2021). Importance of living arrangements and coparenting quality for young children's mental health after parental divorce: A cross-sectional parental survey. BMJ Paediatrics Open, 5(1), e000657

M. Broberg. (2012). Young children's well-being in Finnish stepfamilies. Early Child Development and Care, 182(3), 401-415

M. Frisch and A. Hviid. (2006). Childhood family correlates of heterosexual and homosexual marriages: a national cohort study of two million Danes. Archives of sexual behavior, 35(5), 533-547

M. Gahler, Y. Hong and E. Bernhardt. (2009). Parental Divorce and Union Disruption Among Young Adults in Sweden. JOURNAL OF FAMILY ISSUES, 30(5), 688-713

M. Irhammar and H. Bengtsson. (2004). Attachment in a Group of Adult International Adoptees. Adoption Quarterly, 8(2), 1-25

M. Issakainen and V. Hanninen. (2016). Young people's narratives of depression. Journal of Youth Studies, 19(2), 237-250

M. J. Carr, P. L. H. Mok, S. Antonsen, C. B. Pedersen and R. T. Webb. (2020). Self-harm and violent criminality linked with parental death during childhood. Psychological Medicine, 50(7), 1224-1232

M. J. Carr, S. Steeg, P. L. H. Mok, C. B. Pedersen, S. Antonsen, N. Kapur and R. T. Webb. (2020). Adverse childhood experiences and risk of subsequently engaging in self-harm and violence towards other people-"Dual harm". International Journal of Environmental Research and Public Health, 17(24), 1-13

M. J. Uddin, C. T. Ekstrøm, N. Hemager, C. A. J. Christiani, M. Gregersen, D. V. Ellersgaard, K. S. Spang, A. Greve, B. K. Burton, A. Søndergaard, R. Nudel, P. B. Mortensen, M. G. Pedersen, C. B. Pedersen, Y. Wang, T. Werge, J. Bybjerg-Grauholm, K. J. Von Plessen, V. Bliksted, O. Mors, A. A. E. Thorup and M. Nordentoft. (2021). Is the Association between Parents' Mental Illness and Child Psychopathology Mediated via Home Environment and Caregiver's Psychosocial Functioning? A Mediation Analysis of the Danish High Risk and Resilience Study-VIA7, a Population-Based Cohort Study. Schizophrenia Bulletin Open, 2(1)

M. Jaaskelainen, M. Holmila, I.-L. Notkola and K. Raitasalo. (2016). Mental disorders and harmful substance use in children of substance abusing parents: A longitudinal register-based study on a complete birth cohort born in 1991. Drug and alcohol review, 35(6), 728-740

M. Kontu, H. Hakko, K. Riala and P. Riipinen. (2021). Adolescence Predictors for Drug Crime Offending: A Follow-up Study of Former Adolescent Psychiatric Inpatients. COMMUNITY MENTAL HEALTH JOURNAL, 57(4), 736-745

M. Lasgaard, C. Armour, R. Bramsen and L. Goossens. (2016). Major Life Events as Predictors of Loneliness in Adolescence. Journal of Child & Family Studies, 25(2), 631-637

M. Lowery Wilson, O. Tenovuo, M. Gissler and S. Saarijarvi. (2019). Association between parent mental health and paediatric TBI: epidemiological observations from the 1987 Finnish Birth Cohort. Injury prevention : journal of the International Society for Child and Adolescent Injury Prevention, 25(4), 283-289

M. Lytje. (2018). Voices that want to be heard: Using bereaved Danish students suggestions to update school bereavement response plans. Death Studies, 42(4), 254-267

M. Merikukka, S. Rasanen, H. Hakko, T. Ristikari, M. Gissler and M. Niemela. (2020). Association between parental hospital-treated somatic illnesses in childhood and later mental disorders among offspring up to early adulthood: An explorative study in the 1987 Finnish Birth Cohort. Scandinavian journal of public health, 48(2), 214-223

M. Merikukka, T. Ristikari, A. Tuulio-Henriksson, M. Gissler and M. Laaksonen. (2018). Childhood determinants for early psychiatric disability pension: A 10-year follow-up study of the 1987 Finnish Birth Cohort. The International journal of social psychiatry, 64(8), 715-725

M. N. Christoffersen and K. Soothill. (2003). The long-term consequences of parental alcohol abuse: A cohort study of children in Denmark. Journal of Substance Abuse Treatment, 25(2), 107-116

M. N. Christoffersen, H. D. Poulsen and A. Nielsen. (2003). Attempted suicide among young people: Risk factors in a prospective register based study of Danish children born in 1966. Acta Psychiatrica Scandinavica, 108(5), 350-358

M. N. Christoffersen, K. Soothill and B. Francis. (2008). Risk factors for a first-time drink-driving conviction among young men: A birth cohort study of all men born in Denmark in 1966. Journal of Substance Abuse Treatment, 34(4), 415-425

M. Novak, C. Ahlgren and A. Hammarstrom. (2007). Inequalities in smoking: Influence of social chain of risks from adolescence to young adulthood: A prospective population-based cohort study. International Journal of Behavioral Medicine, 14(3), 181-187

M. P. T. Sumanen, M. J. Koskenvuo, L. H. Sillanmaki and K. J. Mattila. (2009). Peptic ulcer and childhood adversities experienced by working-aged people. World Journal of Gastroenterology, 15(27), 3405-3410

M. Pelkonen, M. Marttunen, J. Kaprio, T. Huurre and H. Aro. (2008). Adolescent risk factors for episodic and persistent depression in adulthood. A 16-year prospective follow-up study of adolescents. Journal of Affective Disorders, 106(1), 123-131

M. Pelkonen, M. Marttunen, M. Henriksson and J. Lonnqvist. (2007). Adolescent adjustment disorder: Precipitant stressors and distress symptoms of 89 outpatients. European Psychiatry, 22(5), 288-295

M. Pierce, K. M. Abel, J. Muwonge, Jr., S. Wicks, A. Nevriana, H. Hope, C. Dalman and K. Kosidou. (2020). Prevalence of parental mental illness and association with socioeconomic adversity among children in Sweden between 2006 and 2016: a population-based cohort study. The Lancet. Public health, 5(11), e583-e591

M. Rostila and J. M. Saarela. (2011). Time does not heal all wounds: Mortality following the death of a parent. Journal of Marriage and Family, 73(1), 236-249

M. Rostila, L. Berg, A. Arat, B. Vinnerljung and A. Hjern. (2016). Parental death in childhood and self-inflicted injuries in young adults-a national cohort study from Sweden. European Child & Adolescent Psychiatry, 25(10), 1103-1111

M. Rytila-Manninen, N. Lindberg, H. Haravuori, K. Kettunen, M. Marttunen, M. Joukamaa and S. Frojd. (2014). Adverse childhood experiences as risk factors for serious mental disorders and inpatient hospitalization among adolescents. Child Abuse & Neglect, 38(12), 2021-2032

M. S. Aaskoven, T. Kjaer and D. Gyrd-Hansen. (2022). Effects of parental health shocks on children's school achievements: A register-based population study. Journal of health economics, 81, 102573

M. Shevlin, E. McElroy, M. N. Christoffersen, A. Elklit, P. Hyland and J. Murphy. (2016). Social, familial and psychological risk factors for psychosis: A birth cohort study using the Danish Registry System. Psychosis: Psychological, Social and Integrative Approaches, 8(2), 95-105

M. Shevlin, P. Hyland, A. Elklit, J. Murphy, S. Murphy, M. Kearney, D. Christie and M. Christoferson. (2019). Social, familial and psychological risk factors for endocrine, nutritional and metabolic disorders in childhood and early adulthood: A birth cohort study using the Danish registry system. Current Psychology: A Journal for Diverse Perspectives on Diverse Psychological Issues, 38(5), 1297-1303

M. Sumanen, A. Rantala, L. H. Sillanmaki and K. J. Mattila. (2007). Childhood adversities experienced by working-age migraine patients. Journal of Psychosomatic Research, 62(2), 139-143

M. Thastum, M. B. Johansen, L. Gubba, L. B. Olesen and G. Romer. (2008). Coping, social relations, and communication: A qualitative exploratory study of children of parents with cancer. Clinical Child Psychology and Psychiatry, 13(1), 123-138

M. V. Trondsen and A. Tjora. (2014). Communal normalization in an online self-help group for adolescents with a mentally ill parent. Qualitative Health Research, 24(10), 1407-1417

M. V. Trondsen. (2012). Living with a mentally ill parent: Exploring adolescents' experiences and perspectives. Qualitative Health Research, 22(2), 174-188

M. Vuorenmaa, N. Halme, M. Kaunonen, P. Astedt-Kurki and M. L. Perala. (2017). Determinants of maternal and paternal empowerment: exploring the role of childhood adversities. EUROPEAN JOURNAL OF PUBLIC HEALTH, 27(1), 35-41

M. Wadsby, G. Priebe and C. G. Svedin. (2014). Adolescents with alternating residence after parental divorce: A comparison with adolescents living with both parents or with a single parent. Journal of Child Custody: Research, Issues, and Practices, 11(3), 202-215

M. Wallinius, C. Delfin, E. Billstedt, T. Nilsson, H. Anckarsater and B. Hofvander. (2016). Offenders in emerging adulthood: School maladjustment, childhood adversities, and prediction of aggressive antisocial behaviors. Law and human behavior, 40(5), 551-563

M. Widemalm and F. Hjarthag. (2015). The forum as a friend: parental mental illness and communication on open Internet forums. Social psychiatry and psychiatric epidemiology, 50(10), 1601-1607

M. Ystgaard, I. Hestetun, M. Loeb and L. Mehlum. (2004). Is there a specific relationship between childhood sexual and physical abuse and repeated suicidal behavior? Child Abuse & Neglect, 28(8), 863-875

N. Bilenberg, C. S. Rasmussen, L. G. Nielsen, E. Christiansen and D. J. Petersen. (2013). Adverse life events as risk factors for behavioral and emotional problems in a seven-year follow up of a population-based child cohort. European Child and Adolescent Psychiatry, 22(2), S263

N. H. Rod, J. Bengtsson, E. Budtz-Jorgensen, C. Clipet-Jensen, D. Taylor-Robinson, A. M. N. Andersen, N. Dich and A. Rieckmann. (2020). Trajectories of childhood adversity and mortality in early adulthood: a population-based cohort study. The Lancet, 396(10249), 489-497

N. H. Rod, J. Bengtsson, L. K. Elsenburg, D. Taylor-Robinson and A. Rieckmann. (2021). Hospitalisation patterns among children exposed to childhood adversity: a population-based cohort study of half a million children. The Lancet Public Health, 6(11), e826-e835

N. Kerekes, B. Zouini, S. Tingberg and S. Erlandsson. (2021). Psychological Distress, Somatic Complaints, and Their Relation to Negative Psychosocial Factors in a Sample of Swedish High School Students. Frontiers in public health, 9, 669958

N. M. Nielsen, A. V. Hansen, J. Simonsen and A. Hviid. (2012). Stressful life events in childhood and risk of infectious disease hospitalization. European journal of pediatrics, 171(1), 173-9

N. M. Nielsen, B. V. Pedersen, E. Stenager, N. Koch-Henriksen and M. Frisch. (2014). Stressful life-events in childhood and risk of multiple sclerosis: A Danish nationwide cohort study. Multiple Sclerosis Journal, 20(12), 1609-1615

O. Heradstveit, B. S. M. Haugland, S. A. Nilsen, T. Boe, B. Sivertsen and M. Hysing. (2021). Parental mental illness as a risk factor for adolescent psychiatric disorders: A registry-based study of specialized child and adolescent health services. Child & Youth Services, No-Specified

O. Kiviruusu, T. Huurre, H. Aro, M. Marttunen and A. Haukkala. (2015). Self-esteem growth trajectory from adolescence to mid-adulthood and its predictors in adolescence. Advances in life course research, 23, 29-43

O. Koskinen, A. Sauvola, P. Valonen, H. Hakko, M. R. Järvelin and P. Räsänen. (2001). Increased risk of violent recidivism among adult males is related to single-parent family during childhood: The northern finland 1966 birth cohort study. Journal of Forensic Psychiatry, 12(3), 539-548

O. Kravdal and E. Grundy. (2019). Children's age at parental divorce and depression in early and mid-adulthood. Population studies, 73(1), 37-56

P. Hyland, M. Shevlin, A. Elklit, M. Christoffersen and J. Murphy. (2016). Social, familial and psychological risk factors for mood and anxiety disorders in childhood and early adulthood: a birth cohort study using the Danish Registry System. Social psychiatry and psychiatric epidemiology, 51(3), 331-338

P. L. H. Mok, C. B. Pedersen, D. Springate, A. Astrup, N. Kapur, S. Antonsen, O. Mors and R. T. Webb. (2016). Parental Psychiatric Disease and Risks of Attempted Suicide and Violent Criminal Offending in Offspring A Population-Based Cohort Study. JAMA PSYCHIATRY, 73(10), 1015-1022

P. Martikainen, K. Korhonen, H. Moustgaard, M. Aaltonen and H. Remes. (2018). Substance abuse in parents and subsequent risk of offspring psychiatric morbidity in late adolescence and early adulthood: A longitudinal analysis of siblings and their parents. Social Science and Medicine, 217, 106-111

P. Nylander, A. Kallstrom and K. Hellfeldt. (2018). After a childhood with a parent in prison - relationships and well-being as a child and young adult. International journal of prisoner health, 14(1), 34-45

P. Polkki, S.-A. Ervast and M. Huupponen. (2004). Coping and Resilience of Children of a Mentally Ill Parent. Social Work in Health Care, 39(1), 151-163

P. Vidal-Ribas, A. Stringaris, C. Rück, E. Serlachius, P. Lichtenstein and D. Mataix-Cols. (2015). Are stressful life events causally related to the severity of obsessive-compulsive symptoms? A monozygotic twin difference study. European Psychiatry, 30(2), 309-316

P. Welford, A. K. Danielsson and H. Manhica. (2022). Parental substance use disorder and offspring not in education, employment or training: A national cohort study of young adults in Sweden. Addiction (Abingdon, England)

R. Chen, A. Regodon Wallin, E. Noren Selinus, A. Sjolander, K. Fall, U. Valdimarsdottir, K. Czene and F. Fang. (2018). Psychiatric disorders among children of parents with cancer: A Swedish register-based matched cohort study. Psycho-Oncology, 27(7), 1854-1860

R. Chen, K. Fall, K. Czene, B. Kennedy, U. Valdimarsdottir and F. Fang. (2018). Impact of parental cancer on IQ, stress resilience, and physical fitness in young men. Clinical epidemiology, 10, 593-604

R. Eklund, A. Alvariza, U. Kreicbergs, L. Jalmsell and M. Lovgren. (2020). The family talk intervention for families when a parent is cared for in palliative care - potential effects from minor children's perspectives. BMC palliative care, 19(1), 50

R. F. Olsen. (2020). Paternal imprisonment and adolescent boys’ charge rates. European Journal of Criminology

R. Grahn, M. Padyab and L. Lundgren. (2020). Associations between a risky psychosocial childhood and recurrent addiction compulsory care as adult. Nordic Studies on Alcohol and Drugs, 37(1), 54-68

R. Isohookana, M. Marttunen, H. Hakko, P. Riipinen and K. Riala. (2016). The impact of adverse childhood experiences on obesity and unhealthy weight control behaviors among adolescents. Comprehensive Psychiatry, 71, 17-24

R. Kaltiala-Heino, M. Rimpela, P. Rantanen and P. Laippala. (2001). Adolescent depression: the role of discontinuities in life course and social support. JOURNAL OF AFFECTIVE DISORDERS, 64(2), 155-166

R. Paananen, A. Tuulio-Henriksson, M. Merikukka and M. Gissler. (2021). Intergenerational transmission of psychiatric disorders: the 1987 Finnish Birth Cohort study. EUROPEAN CHILD & ADOLESCENT PSYCHIATRY, 30(3), 381-389

R. Paananen, T. Ristikari, M. Merikukka and M. Gissler. (2013). Social determinants of mental health: A Finnish nationwide follow-up study on mental disorders. Journal of Epidemiology and Community Health, 67(12), 1025-1031

R. T. Webb, S. Wicks, C. Dalman, A. R. Pickles, L. Appleby, P. B. Mortensen, B. Haglund and K. M. Abel. (2010). Influence of environmental factors in higher risk of sudden infant death syndrome linked with parental mental illness. Archives of general psychiatry, 67(1), 69-77

S. A. Nilsen, K. Breivik, B. Wold, K. G. Askeland, B. Sivertsen, M. Hysing and T. Boe. (2020). Divorce and adolescent academic achievement: Heterogeneity in the associations by parental education. PLoS ONE, 15(3), e0229183

S. B. Laftman, M. Bergstrom, B. Modin and V. Ostberg. (2014). Joint physical custody, turning to parents for emotional support, and subjective health: A study of adolescents in Stockholm, Sweden. Scandinavian journal of public health, 42(5), 456-462

S. D. Stergaard, J. T. Larsen, L. Petersen, G. D. Smith and E. Agerbo. (2019). Psychosocial Adversity in Infancy and Mortality Rates in Childhood and Adolescence: A Birth Cohort Study of 1.5 Million Individuals. Epidemiology, 30(2), 246-255

S. E. Jorgensen, L. C. Thygesen, A. Andersen, P. Due and S. I. Michelsen. (2022). Parental Illness and Life Satisfaction among Young People: A Cross-Sectional Study of the Importance of School Factors. International journal of environmental research and public health, 19(5)

S. E. Jorgensen, L. C. Thygesen, S. I. Michelsen, P. Due, P. E. Bidstrup, B. L. Hoeg and A. Andersen. (2021). Why do some adolescents manage despite parental illness? Identifying promotive factors. Journal of Adolescent Health, 69(2), 335-341

S. F. Reiter, S. Hjorleifsson, H. J. Breidablik and E. Meland. (2013). Impact of divorce and loss of parental contact on health complaints among adolescents. Journal of Public Health (United Kingdom), 35(2), 278-285

S. Frojd, M. Marttunen and R. Kaltiala-Heino. (2012). The effect of adolescent- and parent-induced family transitions in middle adolescence. Nordic Journal of Psychiatry, 66(4), 254-259

S. H. Andersen, L. Steinberg and J. Belsky. (2021). Beyond early years versus adolescence: The interactive effect of adversity in both periods on life-course development. Developmental psychology, 57(11), 1958-1967

S. H. Andersen. (2021). Association of Youth Age at Exposure to Household Dysfunction With Outcomes in Early Adulthood. JAMA Network Open, 4(1), e2032769-e2032769

S. H. Haugland and T. H. Elgan. (2021). Prevalence of parental alcohol problems among a general population sample of 28,047 norwegian adults: Evidence for a socioeconomic gradient. International Journal of Environmental Research and Public Health, 18(10), 5412

S. H. Haugland, A. Dovran, A. U. Albaek and B. Sivertsen. (2021). Adverse Childhood Experiences Among 28,047 Norwegian Adults From a General Population. Frontiers in public health, 9, 711344

S. H. Haugland, B. Carvalho, T. H. Stea, A. Strandheim and J.-K. Vederhus. (2021). Associations between parental alcohol problems in childhood and adversities during childhood and later adulthood: a cross-sectional study of 28047 adults from the general population. Substance abuse treatment, prevention, and policy, 16(1), 47

S. H. Haugland, T. L. Holmen, S. Krokstad, E. R. Sund and G. H. Bratberg. (2015). Intergenerational Hazardous Alcohol Use and Area Factors: The HUNT Study, Norway. Substance use & misuse, 50(14), 1753-1764

S. Helseth and N. Ulfsaet. (2005). Parenting experiences during cancer. Journal of Advanced Nursing (Wiley-Blackwell), 52(1), 38-46

S. Heuckendorff, M. N. Johansen, C. Overgaard, S. P. Johnsen, Y. Kelly and K. Fonager. (2022). Parental mental health, socioeconomic position and the risk of asthma in children-a nationwide Danish register study. European journal of public health, 32(1), 14-20

S. J. Kuramoto, B. Runeson, E. A. Stuart, P. Lichtenstein and H. C. Wilcox. (2013). Time to hospitalization for suicide attempt by the timing of parental suicide during offspring early development. JAMA Psychiatry, 70(2), 149-157

S. J. Kuramoto, E. A. Stuart, B. Runeson, P. Lichtenstein, N. Langstrom and H. C. Wilcox. (2010). Maternal or Paternal Suicide and Offspring's Psychiatric and Suicide-Attempt Hospitalization Risk. PEDIATRICS, 126(5), E1026-E1032

S. J. Latendresse, R. J. Rose, R. J. Viken, L. Pulkkinen, J. Kaprio and D. M. Dick. (2008). Parenting mechanisms in links between parents' and adolescents' alcohol use behaviors. Alcoholism: Clinical and Experimental Research, 32(2), 322-330

S. K. Dahl, J. T. Larsen, L. Petersen, M. B. Ubbesen, P. B. Mortensen, T. Munk-Olsen and K. L. Musliner. (2017). Early adversity and risk for moderate to severe unipolar depressive disorder in adolescence and adulthood: A register-based study of 978,647 individuals. Journal of Affective Disorders, 214, 122-129

S. K. Korpimaki, M. P. T. Sumanen, L. H. Sillanmaki and K. J. Mattila. (2010). Cancer in working-age is not associated with childhood adversities. Acta Oncologica, 49(4), 436-440

S. Kailaheimo-Lonnqvist and S. Kotimaki. (2020). Cause of parental death and child's health and education: The role of parental resources. SSM - Population Health, 11, 100632

S. Kailaheimo-Lonnqvist, A. E. Fasang, M. Jalovaara and E. Struffolino. (2021). Is Parental Divorce Homogamy Associated With a Higher Risk of Separation From Cohabitation and Marriage? Demography, 58(6), 2219-2241

S. Kailaheimo-Lönnqvist and J. Erola. (2020). Child’s age at parental death and university education. European Societies, 22(4), 433-455

S. Kjoelaas, K. H. Tilleras and K. B. Feragen. (2020). The Ripple Effect: A Qualitative Overview of Challenges When Growing Up in Families Affected by Huntington's Disease. Journal of Huntington's disease, 9(2), 129-141

S. M. Volanen, S. Suominen, E. Lahelma, M. Koskenvuo and K. Silventoinen. (2006). Sense of coherence and its determinants: A comparative study of the Finnish-speaking majority and the Swedish-speaking minority in Finland. Scandinavian Journal of Public Health, 34(5), 515-525

S. Meltzer-Brody, J. T. Larsen, L. Petersen, J. Guintivano, A. Di Florio, W. C. Miller, P. F. Sullivan and T. Munk-Olsen. (2018). Adverse life events increase risk for postpartum psychiatric episodes: A population-based epidemiologic study. Depression and Anxiety, 35(2), 160-167

S. Peiponen, E. Laukkanen, V. Korhonen, U. Hintikka and J. Lehtonen. (2006). The association of parental alcohol abuse and depression with severe emotional and behavioural problems in adolescents: A clinical study. International Journal of Social Psychiatry, 52(5), 395-407

S. Rasanen, M. Niemela, T. Nordstrom, H. Hakko, M. Haapea, C. A. Marshall and J. Miettunen. (2019). Parental hospital-treated somatic illnesses and psychosis of the offspring-The Northern Finland Birth Cohort 1986 study. Early Intervention in Psychiatry, 13(2), 290-296

S. Thor, T. Hemmingsson, A.-K. Danielsson and J. Landberg. (2022). Fathers' alcohol consumption and risk of substance-related disorders in offspring. Drug and alcohol dependence, 233, 109354

S. Winqvist, J. Jokelainen, H. Luukinen and M. Hillbom. (2007). Parental alcohol misuse is a powerful predictor for the risk of traumatic brain injury in childhood. Brain Injury, 21(10), 1079-1085

S. Aasen Nilsen, K. Breivik, B. Wold and T. Bøe. (2018). Divorce and Family Structure in Norway: Associations With Adolescent Mental Health. Journal of Divorce and Remarriage, 59(3), 175-194

T. Agerup, S. Lydersen, J. Wallander and A. M. Sund. (2015). Maternal and paternal psychosocial risk factors for clinical depression in a Norwegian community sample of adolescents. Nordic Journal of Psychiatry, 69(1), 35-41

T. Angarne-Lindberg and M. Wadsby. (2009). Fifteen years after parental divorce: Mental health and experienced life-events. Nordic Journal of Psychiatry, 63(1), 32-43

T. Angarne-Lindberg and M. Wadsby. (2011). Sense of Coherence in Young Adults With and Without Experience of Parental Divorce in Childhood. Journal of Divorce & Remarriage, 52(5), 309-321

T. Angarne-Lindberg and M. Wadsby. (2012). Psychiatric and somatic health in relation to experience of parental divorce in childhood. The International journal of social psychiatry, 58(1), 16-25

T. Bylund Grenklo, U. Kreicbergs, A. Hauksdottir, U. A. Valdimarsdottir, T. Nyberg, G. Steineck and C. J. Furst. (2013). Self-injury in teenagers who lost a parent to cancer: a nationwide, population-based, long-term follow-up. JAMA pediatrics, 167(2), 133-40

T. Bylund Grenklo, U. Kreicbergs, U. A. Valdimarsdottir, T. Nyberg, G. Steineck and C. J. Furst. (2014). Self-injury in youths who lost a parent to cancer: nationwide study of the impact of family-related and health-care-related factors. Psycho-oncology, 23(9), 989-97

T. Eide, A. Faugli, E. Kufas, N. H. Mjosund and G. Eilertsen. (2020). Mental health as perceived by Norwegian adolescents living with parental somatic illness: Living in an earthquake zone. International Journal of Qualitative Studies on Health and Well-being, 15(1)

T. H. Lyngstad and H. Engelhardt. (2009). The influence of offspring's sex and age at parents' divorce on the intergenerational transmission of divorce, Norwegian first marriages 1980-2003. Population studies, 63(2), 173-185

T. Hemmingsson, A. K. Danielsson and D. Falkstedt. (2017). Fathers’ alcohol consumption and risk of alcohol-related hospitalization in offspring before 60 years of age. Drugs: Education, Prevention and Policy, 24(1), 3-8

T. Huurre, H. Junkkari and H. Aro. (2006). Long-term psychosocial effects of parental divorce: A follow-up study from adolescence to adulthood. European Archives of Psychiatry and Clinical Neuroscience, 256(4), 256-263

T. Huurre, T. Lintonen, J. Kaprio, M. Pelkonen, M. Marttunen and H. Aro. (2010). Adolescent risk factors for excessive alcohol use at age 32 years. A 16-year prospective follow-up study. Social psychiatry and psychiatric epidemiology, 45(1), 125-134

T. Korhonen, A. C. Huizink, D. M. Dick, L. Pulkkinen, R. J. Rose and J. Kaprio. (2008). Role of individual, peer and family factors in the use of cannabis and other illicit drugs: A longitudinal analysis among Finnish adolescent twins. Drug and Alcohol Dependence, 97(1), 33-43

T. Lund, J. H. Andersen, T. N. Winding, K. Biering and M. Labriola. (2013). Negative life events in childhood as risk indicators of labour market participation in young adulthood: A prospective birth cohort study. PLoS ONE, 8(9), 0075860

T. Laajasalo and H. Hakkanen. (2004). Background characteristics of mentally ill homicide offenders - A comparison of five diagnostic groups. Journal of Forensic Psychiatry and Psychology, 15(3), 451-474

T. Makikyro, A. Sauvola, J. Moring, J. Veijola, P. Nieminen, M.-R. Jarvelin and M. Isohanni. (1998). Hospital-treated psychiatric disorders in adults with a single-parent and two-parent family background: A 28-year follow-up of the 1966 Northern Finland Birth Cohort. Family Process, 37(3), 335-344

T. Ristkari, A. Sourander, J. Ronning, G. Nikolakaros and H. Helenius. (2008). Life events, self-reported psychopathology and sense of coherence among young men - A population-based study. Nordic Journal of Psychiatry, 62(6), 464-471

T. Ruud, D. Maybery, A. Reupert, B. Weimand, K. Foster, A. Grant, B. E. Skogoy and S. O. Ose. (2019). Adult mental health outpatients who have minor children: Prevalence of parents, referrals of their children, and patient characteristics. Frontiers in Psychiatry, 10

T. Skardhamar. (2009). Family Dissolution and Children's Criminal Careers. EUROPEAN JOURNAL OF CRIMINOLOGY, 6(3), 203-223

U. K. Tulisalo and H. M. Aro. (2000). Paternal remarriage as a modifier of proneness to depression in young adulthood. Journal of Affective Disorders, 57(1), 179-184

U. Mustonen, T. Huurre, O. Kiviruusu, A. Haukkala and H. Aro. (2011). Long-Term Impact of Parental Divorce on Intimate Relationship Quality in Adulthood and the Mediating Role of Psychosocial Resources. Journal of Family Psychology, 25(4), 615-619

V. Bergink, J. T. Larsen, M. H. Hillegers, S. K. Dahl, H. Stevens, P. B. Mortensen, L. Petersen and T. Munk-Olsen. (2016). Childhood adverse life events and parental psychopathology as risk factors for bipolar disorder. Translational psychiatry, 6(10), e929

V. Ostensjo, K. Moen, T. Storesund and A. Rosen. (2017). Prevalence of Painful Temporomandibular Disorders and Correlation to Lifestyle Factors among Adolescents in Norway. Pain Research and Management, 2017, 2164825

W. Pedersen. (2001). Adolescent victims of violence in a welfare state. British Journal of Criminology, 41(1), 1-21

W. Sigle-Rushton, T. H. Lyngstad, P. L. Andersen and O. Kravdal. (2014). Proceed with caution? Parents' union dissolution and children's educational achievement. Journal of Marriage and Family, 76(1), 161-174
